# A comprehensive study of the genetic impact of rare variants in *SORL1* in European early-onset Alzheimer’s disease

**DOI:** 10.1007/s00401-016-1566-9

**Published:** 2016-03-30

**Authors:** Jan Verheijen, Tobi Van den Bossche, Julie van der Zee, Sebastiaan Engelborghs, Raquel Sanchez-Valle, Albert Lladó, Caroline Graff, Håkan Thonberg, Pau Pastor, Sara Ortega-Cubero, Maria A. Pastor, Luisa Benussi, Roberta Ghidoni, Giuliano Binetti, Jordi Clarimon, Alberto Lleó, Juan Fortea, Alexandre de Mendonça, Madalena Martins, Oriol Grau-Rivera, Ellen Gelpi, Karolien Bettens, Ligia Mateiu, Lubina Dillen, Patrick Cras, Peter P. De Deyn, Christine Van Broeckhoven, Kristel Sleegers

**Affiliations:** Neurodegenerative Brain Diseases Group, Department of Molecular Genetics, VIB, University of Antwerp, CDE Universiteitsplein 1, 2610 Antwerp, Belgium; Institute Born-Bunge, University of Antwerp, Antwerp, Belgium; Department of Neurology, Antwerp University Hospital, Edegem, Belgium; Department of Neurology and Memory Clinic, Hospital Network Antwerp (ZNA) Middelheim and Hoge Beuken, Antwerp, Belgium; Alzheimer’s Disease and Other Cognitive Disorders Unit, Neurology Department, Hospital Clínic, Institut d’Investigacions Biomediques August Pi i Sunyer (IDIBAPS), Barcelona, Spain; Department of Neurobiology, Care Sciences and Society (NVS), Center for Alzheimer Research, Division of Neurogeriatrics, Karolinska Institutet, Huddinge, Sweden; Genetics Unit, Department of Geriatric Medicine, Karolinska University Hospital, Stockholm, Sweden; Memory Unit, Department of Neurology, University Hospital Mútua de Terrassa, University of Barcelona School of Medicine, Terrassa, Barcelona Spain; Centro de Investigación Biomédica en Red de Enfermedades Neurodegenerativas (CIBERNED), Instituto de Salud Carlos III, Madrid, Spain; Department of Neurology, Complejo Asistencial Universitario de Palencia, Palencia, Spain; Neuroimaging Laboratory, Division of Neurosciences, Center for Applied Medical Research (CIMA), University of Navarra, Pamplona, Spain; Department of Neurology, Clínica Universidad de Navarra, University of Navarra School of Medicine, Pamplona, Spain; Molecular Markers Laboratory, Istituto di Ricovero e Cura a Carattere Scientifico (IRCCS), Istituto Centro San Giovanni di Dio-Fatebenefratelli, Brescia, Italy; MAC Memory Center, Istituto di Ricovero e Cura a Carattere Scientifico (IRCCS), Istituto Centro San Giovanni di Dio-Fatebenefratelli, Brescia, Italy; Department of Neurology, IIB Sant Pau, Hospital de la Santa Creu i Sant Pau, Universidad Autònoma de Barcelona, Barcelona, Spain; Faculty of Medicine and Institute of Molecular Medicine, University of Lisbon, Lisbon, Portugal; Neurological Tissue Bank of the Biobanc, Hospital Clinic, Institut d’Investigacions Biomediques August Pi i Sunyer (IDIBAPS), Barcelona, Spain; Bioinformatics Unit, Department of Molecular Genetics, VIB, Antwerp, Belgium

**Keywords:** *SORL1*, Haploinsufficiency, Loss-of-function, Rare variants, Alzheimer, Early onset, Meta-analysis

## Abstract

The sortilin-related receptor 1 (*SORL1*) gene has been associated with increased risk for Alzheimer’s disease (AD). Rare genetic variants in the *SORL1* gene have also been implicated in autosomal dominant early-onset AD (EOAD). Here we report a large-scale investigation of the contribution of genetic variability in *SORL1* to EOAD in a European EOAD cohort. We performed massive parallel amplicon-based re-sequencing of the full coding region of *SORL1* in 1255 EOAD patients and 1938 age- and origin-matched control individuals in the context of the European Early-Onset Dementia (EOD) consortium, originating from Belgium, Spain, Portugal, Italy, Sweden, Germany, and Czech Republic. We identified six frameshift variants and two nonsense variants that were exclusively present in patients. These mutations are predicted to result in haploinsufficiency through nonsense-mediated mRNA decay, which could be confirmed experimentally for *SORL1* p.Gly447Argfs*22 observed in a Belgian EOAD patient. We observed a 1.5-fold enrichment of rare non-synonymous variants in patients (carrier frequency 8.8 %; SkatOMeta *p* value 0.0001). Of the 84 non-synonymous rare variants detected in the full patient/control cohort, 36 were only detected in patients. Our findings underscore a role of rare *SORL1* variants in EOAD, but also show a non-negligible frequency of these variants in healthy individuals, necessitating the need for pathogenicity assays. Premature stop codons due to frameshift and nonsense variants, have so far exclusively been found in patients, and their predicted mode of action corresponds with evidence from in vitro functional studies of SORL1 in AD.

**Electronic supplementary material:**

The online version of this article (doi:10.1007/s00401-016-1566-9) contains supplementary material, which is available to authorized users.

## Introduction

Alzheimer disease (AD) is the most common neurodegenerative disease and the predominant cause of dementia worldwide. Up to 10 % of AD patients is diagnosed with early-onset AD (EOAD), manifesting symptoms before the age of 65 years [18]. EOAD has a very strong genetic component, with a heritability estimate of 92–100 % [53]. A positive family history of AD is present in 35–60 % of EOAD patients, with up to 15 % of familial EOAD cases showing autosomal dominant inheritance [8, 19]. However, mutations in the known causal genes, encoding Amyloid Precursor Protein (*APP*), Presenilin 1 and 2 (*PSEN1* and *PSEN2*), explain only 5–10 % of EOAD cases [4, 19, 53].

Rare variants in the sortilin-related receptor (*SORL1*) gene have been shown to contribute to early-onset as well as late-onset familial AD [35, 37, 48]. *SORL1* was originally identified as a risk gene for AD in a candidate-gene based association study [42]. Early replication studies showed discrepant findings, possibly due to allelic heterogeneity, locus heterogeneity or lack of statistical power due to small cohort size. Nonetheless, the association was subsequently confirmed in meta-analyses [20, 24, 39, 50] and in genome-wide association studies (GWAS) including Korean, Japanese and Caucasian individuals [24, 33, 39]. The protein encoded by *SORL1* is a type-1 transmembrane, mosaic protein showing homology to the vacuolar protein sorting 10 (Vps10p) family, and the lipoprotein receptor-related proteins (LRP) [52]. The protein SORL1 is unique among the Vps10p-family proteins as it contains additional ligand-binding structures within the LRP domains, including a β-propeller domain, a low-density lipoprotein receptor class A domain, and a fibronectin type-3 domain [2, 16]. The SORL1 protein interacts directly with the APP protein through its complement-type repeats within the low-density lipoprotein receptor class A domain [1, 2], and via a six amino acid-stretching FANSHY motif located in the cytoplasmic tail of SORL1 [14]. Interaction with the protein APP, results in sequestering of APP away from the secretase cleavage route, inhibiting formation of the amyloid-β (Aβ) peptide [2, 14, 32, 36]. Functional characterization of downstream effects of variants identified in familial early-onset and late-onset AD patients elucidated a protective role for SORL1 in the amyloidogenic pathway. Investigation of the functional implications of the familial variant, p.Gly511Arg, showed disrupted interaction of the Vps10p domain with amyloid-β monomers, resulting in reduced lysosomal targeting of Aβ peptide by SORL1 [7]. Two additional rare variants, p.Glu270Lys and p.Thr947Met, were reported in familial late-onset AD patients of Caribbean-Hispanic origin. Both increased Aβ1-40 and Aβ1-42 secretion, and APP levels at the cell surface in transfected cell lines [48].

In this study, we investigated the contribution of genetic variants in the *SORL1* coding region to the occurrence of AD in pan-European cohorts of 1255 early-onset AD patients and 1938 age-matched non-affected control individuals.

## Materials and methods

### Study population

The cohort under study consisted of 1255 EOAD patients originating from Flanders-Belgium (*n* = 312), Spain (*n* = 342), Portugal (*n* = 106), Italy (*n* = 205), Sweden (*n* = 183), Germany (*n* = 100), and Czech Republic (*n* = 7), and 1938 age-matched European control individuals originating from Flanders-Belgium (*n* = 748), Spain (*n* = 306), Portugal (*n* = 130), Italy (*n* = 444), Sweden (*n* = 303), and Czech Republic (*n* = 7) (supplementary table 1a). An additional set of patients (*n* = 30), from the same source population, carrying a known pathogenic mutation in *APP*, *PSEN1* or *PSEN2*, were not included in the study cohort, but used for comparison of clinical characteristics. Mean onset age of the patient cohort was 59.0 ± 6.2 years. Mean age at inclusion for the control cohort was 66.4 ± 9.8 years. In both the patient and the control cohort, 60 % was female. In the patient cohort, information on familial history of AD was available for 759 (60 %) individuals. A positive familial history (defined as presence of at least one first-degree relative with AD) was present for 327 (43 %) individuals, while 432 (57 %) individuals were considered sporadic patients. DNA and medical/demographic information on patients and control individuals from Spain, Portugal, Italy, Sweden, Germany, and Czech Republic was ascertained through the EU EOD consortium as previously described (details are provided in supplementary table 1b) [5, 11, 45, 46]. Consensus diagnosis of possible, probable or definite AD was given according to the National Institute of Neurological and Communicative Disorders and Stroke-Alzheimer Disease and Related Disorders Association (NINCDS-ADRDA) [29] and/or the National Institute on Aging-Alzheimer’s Association (NIA-AA) diagnostic criteria [17, 30]. Belgian patients were ascertained at the memory clinics of Middelheim and Hoge Beuken, Hospital Network Antwerp (ZNA), Antwerp [12], and the University Hospitals of Leuven (UHL), Leuven [30]. Belgian control individuals were either recruited from partners of patients and screened for neurological or psychiatric antecedents or neurological complaints or organic disease involving the central nervous system, or community-recruited control individuals who were included after interview concerning medical and familial history and cognitive screening by means of the Mini Mental State Examination (MMSE > 26) [15].

### *SORL1* sequencing

Sequencing of *SORL1* exons 2–48, and at least 15 nt of each exon–intron flanking region, was performed by target enrichment using MASTR technology (Multiplicom, Niel, Belgium). PCR primers flanking each target region were designed using mPCR software (Multiplicom, Niel, Belgium). Target region size for amplification was set at 500 nt. In total, all target regions were covered by 46 amplicons in nine multiplex PCR reactions. Subsequent indexing and sequencing was performed with extension of target-specific primer sequences with universal tag sequences (5′-TCGTCGGCAGCGTCAGATGTGTATAAGAGACAG-Fwd and 5′ GTCTCGTGGGCTCGGAGATGTGTATAAGAGACAG-Rev). Optimal annealing temperature and relative amounts of PCR primers for all targets were established for uniform amplification of each target in the multiplex reaction. Multiplex PCR reactions were performed on 20 ng genomiphied DNA (Illustra GenomiPhi V2; Thermo Fisher, MA, USA). Amplification quality and efficacy was verified by fragment analysis on an ABI 3730 automated sequencer (Applied Biosystems, CA, USA). Subsequently, multiplex PCR amplicons of each individual were pooled to obtain equimolar concentrations of all amplicons. Library purification was performed with AMPureXP beads (Beckman Coulter, CA, USA). Amplicon-specific barcodes (Nextera XT; illumina, CA, USA) were incorporated in a universal PCR step on the pooled libraries. Barcoded samples were subjected to bridge amplification and bead purification prior to sequencing. Sequencing was performed on the Illumina MiSeq platform, using the Illumina reagent kit v2, generating 2 × 250 bp paired-end reads. Trimming of Illumina adapters from raw sequencing Fastq files was performed by Fastq-mcf. Read alignment and mapping was done against whole reference genome hg19 using the Burrows–Wheeler Aligner [26]. Variant calling and annotation was performed using GATK version 2.2 [28] and annotated using the Genomecomb software pipeline [40]. Variants with a read depth below 20 reads or with an imbalanced reference/variant allele read depth exceeding 3:1 were considered false calls. All remaining variants with predicted effect on protein sequence were included in subsequent manual read inspection using the Integrative Genomics Viewer software [41]. In total, 92 % of the *SORL1* target sequence was sequenced at >20× read depth for all included individuals.

Due to high GC content (74 %), *SORL1* exon 1 was sequenced using simplex PCR amplification followed by Sanger sequencing using the BigDye termination cycle sequencing kit v3.1 on the ABI 3730 DNA Analyzer. Sequences were analyzed using Seqman (DNAstar, WI, USA) and NovoSNP software [51]. Rare variant validation was performed on genomic DNA by Sanger sequencing, as was segregation analysis of variant p.Tyr1816Cys. Variant position on genomic level was based on Genbank accession number NC_000011.9, transcript position was based on NM_003105.5, and protein-level position on NP_003096.1.

### In silico prediction

Putative pathogenic effects of coding *SORL1* variants were predicted using Polymorphism Phenotyping software version 2 (PolyPhen2, http://genetics.bwh.harvard.edu/pph2/), Sorting Intolerant from Tolerant (SIFT, http://sift.jcvi.org), SIFTindel for frameshift variants (http://sift-dna.org/), and MutationTaster (http://mutationtaster.org/) databases. Previous identification of variants was investigated by comparison of identified variants against public databases, including the Database of Single Nucleotide Polymorphisms 141 (http://www.ncbi.nlm.nih.gov/SNP/), the Exome Variant Server (http://evs.gs.washington.edu/EVS/), the International HapMap Project (http://hapmancbi.nlm.nih.gov/), the 1000 Genomes Project (http://www.1000genomes.org/), and the Exome Aggregation Consortium database (http://exac.broadinstitute.org/). Protein stability predictions were performed using the FoldX free energy prediction tool [47], implemented within the YASARA molecular graphics suite (http://www.yasara.org/) for missense variants located within the Vps10p domain.

### RNA sequencing

RNA sequencing data were generated for the p.Gly447Argfs*22 frameshift variant carrier. Total RNA was isolated from Epstein–Barr virus immortalized lymphoblast cells derived from whole blood lymphocytes. RNA isolation was performed using 1.0 × 10^7^ lymphoblast cells with the RNeasy mini kit (Qiagen Inc., Valencia, CA, USA) according to manufacturer’s protocol. Depletion of genomic DNA from the RNA sample was performed by turboDNase treatment (Life Technologies, Carlsbad, CA, USA). RNA quality control to determine RNA concentration and RIN value was performed using the Agilent Technologies 2100 Bioanalyzer. RIN value was measured at 9.3 in a concentration of 86 ng/µl total RNA. The sequencing library was constructed using Truseq stranded mRNA Library Prep Reagent Set (Illumina, San Diego, CA, USA). Library preparation was performed using 1 mg total RNA and included poly-A selected RNA extraction, RNA fragmentation, and random-hexamer-primed reverse transcription. Sequencing of prepared libraries was performed using an Illumina HiSeq 2000 sequencer, generating 126,949,218 101-nucleotide paired-end sequence reads. Data analysis was performed using an in-house developed processing pipeline. Removal of read adapters and trimming of read ends was performed using Trimmomatic [3]. Trimmed reads were mapped against the UCSC human reference genome hg19 [43] using the Bowtie short read aligner integrated in Tophat2 [21]. Post-alignment QC and filtering of mapped-reads was performed with RSeqQC [49]. Variant calling was performed by employing GATK [28], VARSCAN [23] and VEP [31] software.

### Nonsense-mediated mRNA decay

Nonsense-mediated mRNA decay (NMD) was investigated for p.Gly447Argfs*22. NMD was inhibited in Epstein–Barr virus immortalized lymphoblast cell lines derived from the p.Gly447Argfs*22 carrier and two non-carrier controls with 150 μg/mL cycloheximide (Sigma, St Louis, MO, USA) at 37 °C for 4 h, as previously described [10]. After incubation, RNA was isolated using the RNeasy mini kit. Depletion of genomic DNA from the RNA sample was performed by turboDNase treatment. Subsequent cDNA synthesis was performed using superscript III first-strand cDNA kit, oligoDT and random hexamers primers (Life Technologies, Carlsbad, CA, USA). Real-time quantitative PCR was performed to investigate the effect of p.Gly447Argfs*22 on *SORL1* expression using SYBR Green technology (Life Technologies, Carlsbad, CA, USA). *SORL1* expression levels were measured in triplicate, with three measurements per experiment in two separate experiments. Expression of *SORL1* in untreated lymphoblast cells was quantified and analyzed with qBasePlus (Biogazelle, Ghent, Belgium). Effect of CHX incubation on *SORL1* expression in the p.Gly447Argfs*22 carrier and two non-carrier controls was quantified using the $2^{-\Delta\Delta \text{C}_{\text{T}}}$ (Livak) method [27].

### Statistical analysis

Low-frequency (MAF between 0.01 and 0.05) and common (MAF ≥ 0.05) *SORL1* coding variants were tested for deviations from Hardy–Weinberg equilibrium using PLINK [38]. Allele frequencies of common and low-frequency variants in patients and controls were compared by *X*^2^ statistics. Odds ratios and 95 % confidence intervals were calculated by logistic regression modeling, corrected for gender and *APOE* ε4 allele carrier status using PLINK. Nominal *p* values were corrected for the number of variants tested using Bonferroni correction. *SORL1* variants with MAF <0.01 were included in rare variant burden analysis for individuals originating from Spain, Italy, Portugal, Sweden, and Belgium. Individuals originating from Czech Republic (7 patients, 7 controls) and Germany (100 patients, 0 controls) were excluded from the analysis based on cohort size. Rare variant burden analysis was performed by collapsing alleles of all rare coding variants across the full *SORL1* coding sequence or separately for each functional protein domain using an optimized sequence kernel association test (SKAT-O test), adjusted for sample size <2000. Rare variants association tests were performed using the R package SeqMeta [9]. SKAT-O meta-analysis was performed using standard beta-weights, and correction for gender and *APOE* ε4 carrier status of included individuals. Presented SKAT-O meta *p* values represent minimal *p* values over *ρ* as proposed by Lee et al. [25]. Correction for multiple testing was performed using Šidák correction. Functional protein domains were determined according to Pottier et al. [37]. Differences in relative lymphoblast *SORL1* expression were calculated using an unpaired nonparametric (Mann–Whitney) test.

## Results

### *SORL1* mutation screening

**Fig. 1:**
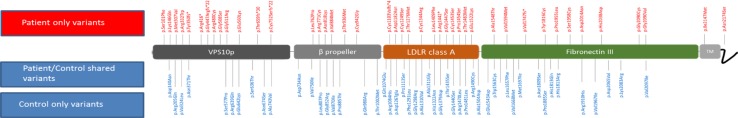
Non-synonymous rare *SORL1* variants identified in EOAD patients and control individuals. Patient-only variants denote variant present in patient cohort. Shared variants denote variants present in both the patient and control cohort. Control-only variants denote variants present in the control cohort. Functional domains are adapted from [37], and based on uniprot information. Protein-level variant position was based on NP_003096. Vps10p vacuolar protein sorting 10 domain, LDLR low-density lipoprotein receptor domain, TM transmembrane domain

We analyzed the coding sequence of *SORL1* in 1255 European early-onset AD patients and 1938 origin-matched control individuals and identified 92 rare frameshift, nonsense and nonsynonymous variants (MAF < 0.01) in a total of 219 individuals, of whom 111 (51 %) were patients (Fig. 1; Supplementary tables 2, 3, 4). In addition, the coding region harbored 102 rare synonymous variants, five low-frequency variants (MAF 0.01–0.05; three missense and two synonymous), one common missense and five common synonymous variants (MAF ≥ 0.05) (Supplementary tables 5, 6).

**Fig. 2:**
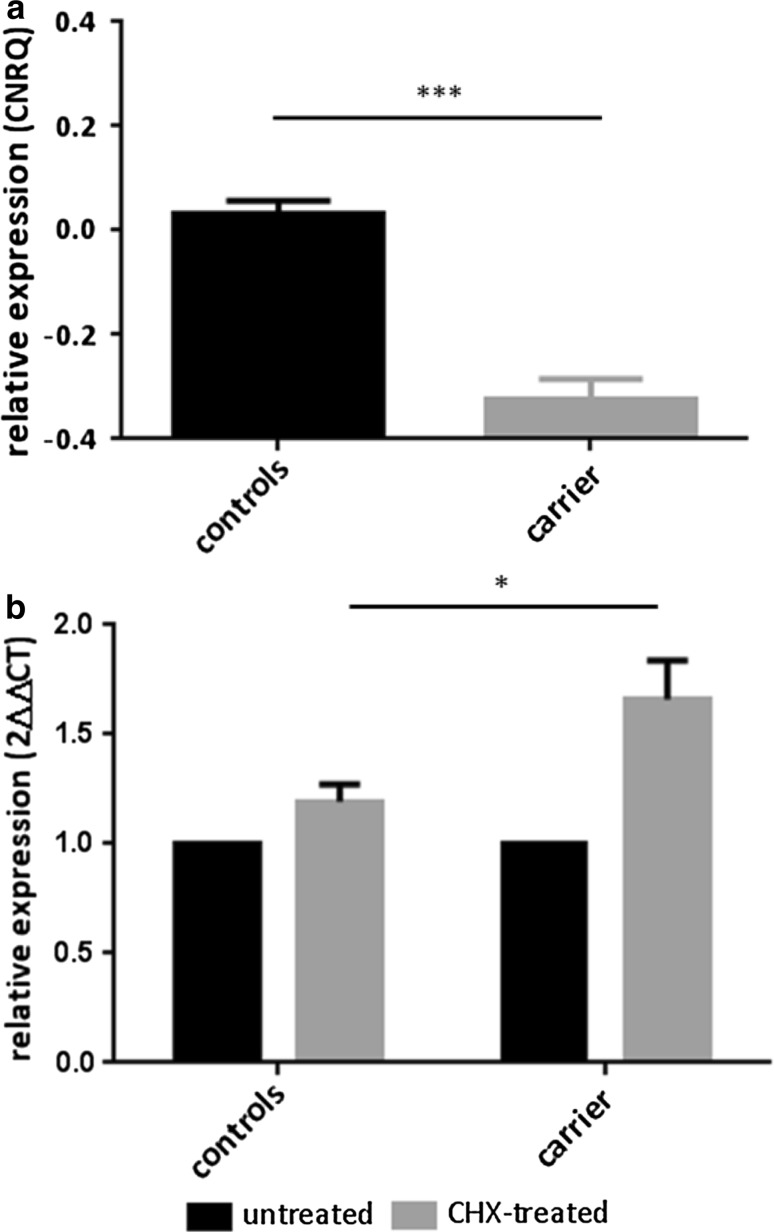
SORL1 expression and investigation of NMD. **a** *SORL1* expression in lymphoblast cell lines of AD patient carrying *SORL1* frameshift variant p.Gly447Argfs*22 and non-carrying control individuals. Measurements per sample were conducted in triplicate, with three measurements per experiment in two separate experiments. *Y-axis* indicates the relative expression quantities of *SORL1*. *Error bars* correspond to the standard error of the mean (SEM). Normalization was carried out against the housekeeping gene *YWHAZ*. Unpaired nonparametric Mann–Whitney test was performed to compare *SORL1* expression of the p.Gly447Argfs*22 variant carrier with the control individuals. **b** SORL1 expression in lymphoblast cell lines of AD patient carrying *SORL1* frameshift variant p.Gly447Argfs*22 and non-carrying controls. *Black bars* represent SORL1 expression in untreated samples (reference, set to 1); *grey bars* represent SORL1 expression after cycloheximide (CHX) treatment (relative to the non-treated sample). *Error bars* correspond to the standard error of the mean (SEM). Unpaired nonparametric Mann–Whitney test was performed to compare the effect of CHX incubation on SORL1 expression of the p.Gly447Argfs*22 variant with the control individuals

The observed rare variants included eight mutations introducing a premature termination codon (PTC): frameshift mutations p.Thr659Serfs*30, p.Cys752Serfs*21, p.Tyr350fs*, p.Gly447Argfs*22, p.Cys1103Valfs*4, p.Val1747fs*, and nonsense variants p.Arg416* and p.Arg1442* (Table 1), predicted to result in haploinsufficiency due to NMD. All PTC mutations were private variants and exclusive to the patient cohort [8/1255 (0.64 %) patients vs. 0/1938 controls]. For one of the frameshift mutation carriers, p.Gly447Argfs*22, biomaterials were available for investigation of the predicted mRNA decay. RNA sequencing on lymphoblast cells demonstrated that the alternative allele (insertion of A) was called, but only in a minority of reads (6.8 %) compared to the reference allele. Quantitative RT-PCR on lymphoblast cells showed reduced *SORL1* expression levels in the p.Gly447Argfs*22 variant carrier compared to non-carrying control individuals (Mann–Whitney *p* value <0.001) (Fig. 2a). Blocking of nonsense-mediated decay by CHX treatment showed significant increase of *SORL1* expression in the p.Gly447Argfs*22 carrier compared to non-carrying control individuals (Mann–Whitney *p* value 0.03) (Fig. 2b).

**Table 1:** Premature termination codon variants identified in EOAD patients

Genomic level nomenclature	cDNA-level nomenclature	Protein-level nomenclature	Protein domain	Country of origin	Gender	Clinical diagnosis	Family history	AAO (in years)	DD (in years)
g.61958_61964delCATCGCAG	c.1050_1057del CATCGCAG	p.Tyr350fs*	Vps10p	Spain	f	Probable AD	S	65	≥2
g.68489C>T	c.1246C>T	p.Arg416*	Vps10p	Sweden	f	Probable AD	U	55	U
g.68579_68579insA	c.1338insA	p.Gly447Argfs*22	Vps10p	Belgium	m	Probable AD	F	64	16
g.93142_93148delCCCCATG	c.1966_1972delCCCCATG	p.Thr659Serfs*30	Vps10p	Spain	f	Probable AD	S	53	≥3
g.98455_98457delCT	c.d2253_2254delCT	p.Cys752Serfs*21	Vps10p	Italy	f	Probable AD	F	50	≥1
g.118037_118037delC	c.3306delC	p.Cys1103Valfs*4	LDLR class A	Spain	f	Probable AD	F	58	≥5
g.138909C>T	c.4324C>T	p.Arg1442*	LDLR class A	Portugal	f	Probable AD	F	60	6
g.155974_155974delG	c.5241delG	p.Val1747 fs*	Fibronectin type III	Belgium	f	Probable AD	F	64	≥6

Functional domains are adapted from [37], and based on uniprot information. Genomic DNA level was based on NC_000011.9, cDNA-level nomenclature was based on NM_003105 according to hg19/GRCh37. Protein-level nomenclature was based on NP_003096

*F* familial, *S* sporadic, *U* unknown, *Del* deletion, *ins* insertion, *AAO* age at onset, *DD* disease duration

In addition to these 8 PTC mutations, we observed 84 rare missense variants in the patient/control cohort. Of the total identified rare missense variants and PTC mutations, 44 (48 %) were only observed in the patient cohort (supplementary table 2). In addition, 19 rare missense variants (21 %) were present in both patient and controls, and 29 variants (32 %) were only observed in controls (supplementary tables 3, 4). One patient carried a frameshift (p.Cys1103Valfs*4) and a missense variant (p.Asp2065Val); three patients and two controls carried double missense variants. Of the rare variants observed in this study, 30 (33 %) were not previously reported in any of the screened databases, the majority of which [22 (73 %)] were only observed in the patient cohort, while seven (23 %) were only found in controls, and one novel variant was detected in patients as well as controls (supplementary tables 2, 3, and 4). Of the variants only observed in the patient cohort, 35 of 44 (80 %) were predicted to be pathogenic by at least two of three prediction tools, whereas 20 of 29 (69 %) variants only observed in the control cohort, and 12 of 19 (63 %) shared variants, were predicted pathogenic (supplementary tables 2, 3, and 4). The effect of variants on protein stability could be modeled for eight variants observed in patients only, and seven variants observed in controls only located in the VPS10p domain. Predicted destabilization (∆∆G-value above 1.0) was shown for four out of eight patient-only variants against one out of seven control-only variants (supplementary table 7).

### Clinicopathological characteristics

**Fig. 3:**
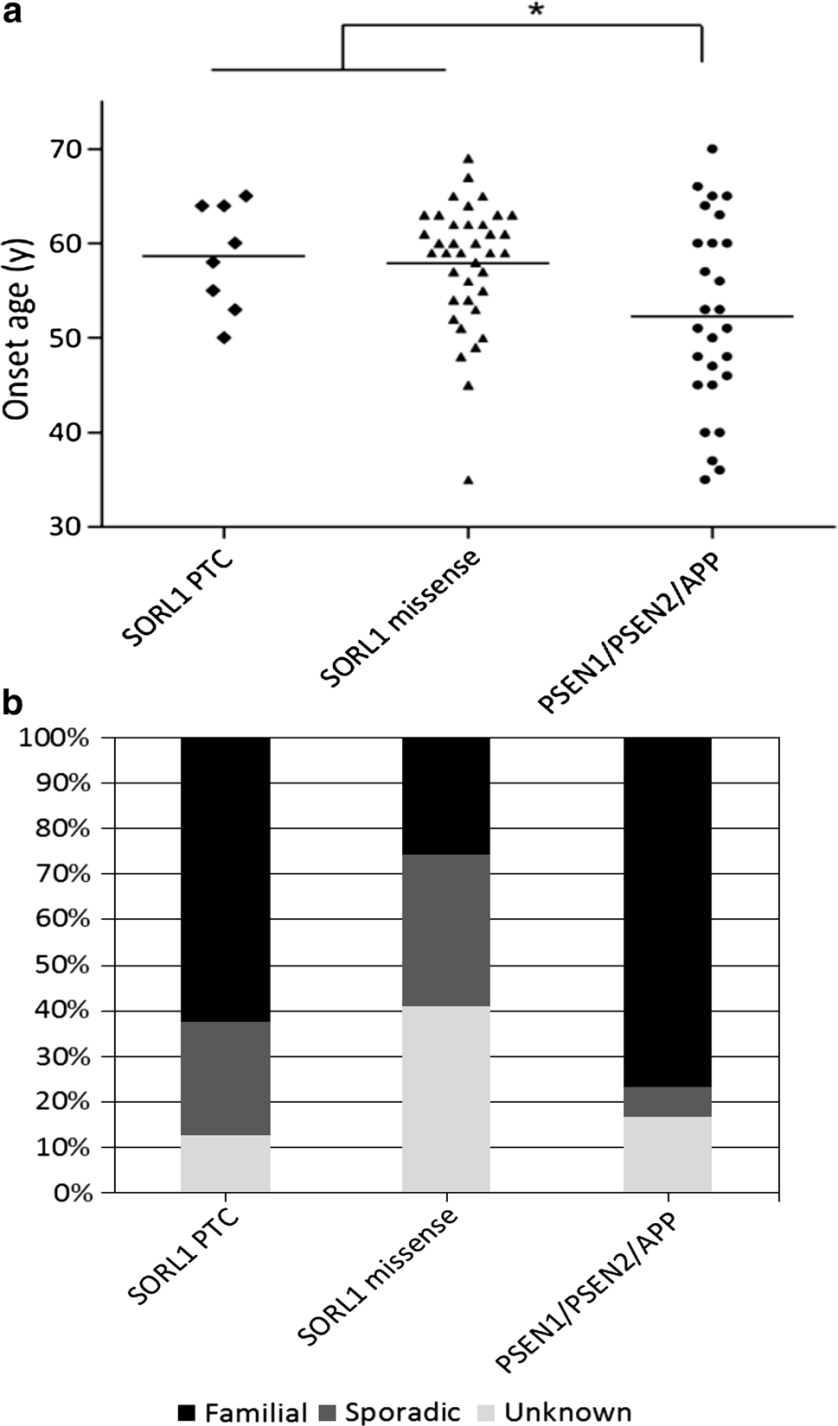
Clinical characteristics of mutation carriers. **a** Scatter plot showing the onset ages for the *SORL1* PTC and patient exclusive missense carriers versus those of *PSEN1*, *PSEN2* and *APP* carriers. Mann–Whitney *U* test *p* value 0.016. **b** The proportion of *SORL1* PTC, *SORL1* missense and *PSEN1*, *PSEN2* and *APP* carriers with a sporadic, unknown or positive familial history for AD

All patients carrying a PTC (*n* = 8) or a patient-only missense (*n* = 39) variant, received a probable (*n* = 44) or definite (*n* = 3) AD diagnosis. The mean onset age (OA) of the PTC carriers was 58.6 ± 5.2 years, with an age range of 15 (50–65) years, and a mean disease duration of 11.0 ± 5.0 years (Fig. 3a). The missense carriers had a mean OA of 57.9 ± 6.5 years, with a wide age range of 34 (35–69) years, and mean disease duration of 12.5 ± 5.5 years. In comparison, the mean OA was 52.4 ± 10.9 years for *PSEN1* carriers (*n* = 23), 49.5 ± 1.5 years for *PSEN2* carriers (*n* = 2) and 53.0 ± 6.8 years for *APP* carriers (*n* = 5) in the EOD cohort. A positive familial history was reported in 71.4 % (5/7) of the *SORL1* PTC carriers, and in 43.5 % (10/23) of the *SORL1* missense carriers. For the *PSEN1* carriers a familial history was reported in 88.9 % (16/18), and in 100 % of the *PSEN2* (2/2) and *APP* (5/5) carriers (Fig. 3b). For one variant, located in the fibronectin type III domain (p.Tyr1816Cys), DNA of relatives was available. The variant was also present in an affected sister, and not present in an unaffected sister (supplementary figure 1).

Additional clinical information was available for 6 PTC carriers. All presented with an insidious memory dysfunction. In one carrier (p.Cys752Serfs*21), disease onset was also accompanied by apathy. Further progression of disease in the carriers was typical of AD, with progression to a global cognitive deterioration and functional dependence. Of note, in patient DR12.1 (p.Gly447Argfs*22), the onset of visual hallucinations, a fluctuating extrapyramidal syndrome and a REM-sleep behavior disorder, after a disease duration of 11 years, led to the suspicion of a concomitant Lewy body pathology.

**Fig. 4:**
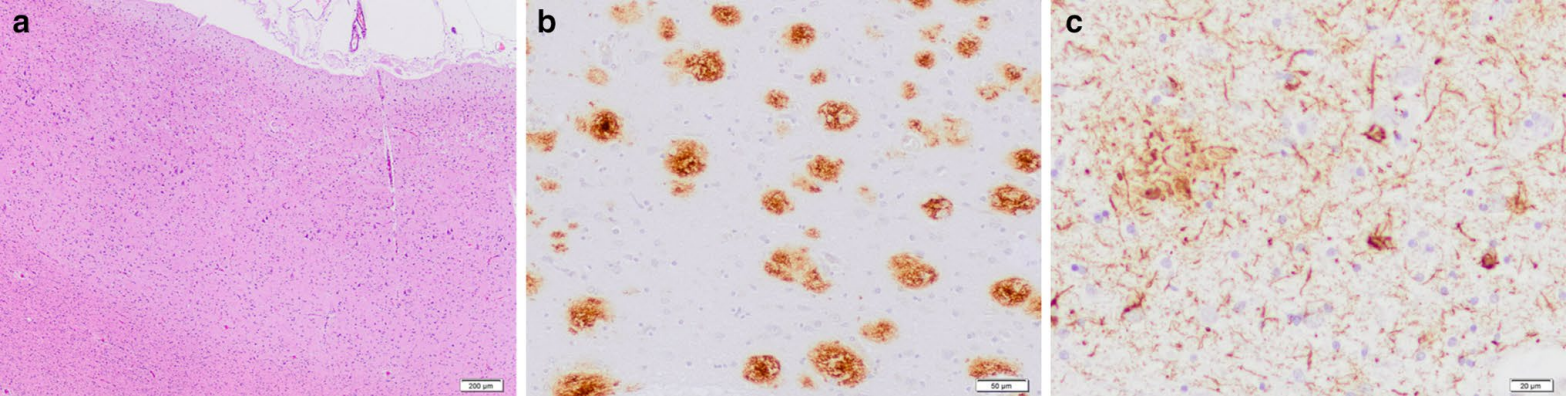
Neuropathology of *SORL1* missense carrier CS540. Neuropathological brain examination of a *SORL1* missense carrier showing cortical thinning and superficial spongiosis in the frontal cortex, where pyramidal neurons contain very large tangles and abundant lipofuscin (**a**). Frequent mature and in a lesser extent diffuse beta-amyloid plaques are observed in the neocortical regions (**b**), as well as the cingulum and hippocampus. Frequent hyperphosphorylated tau immunoreactive (AT8) threads and large globose neurofibrillary tangles are present in neocortical areas (**c**) and cingulum

Neuropathological examination was not available for *SORL1* PTC carriers, but has been performed in 3 *SORL1* missense carriers [DR112.1 (p.Leu762Pro), CS540 (p.Ala1548Thr) and CS770 (p.Gly1447Ser)]. All three had high-level AD neuropathologic changes (A3B3C3) [34], confirming the clinical AD diagnosis. Neuronal loss, gliosis and abnormal protein deposition—mostly in the form of senile plaques and neurofibrillary tangles (Fig. 4)—were most pronounced in the neocortical areas, amygdala, hippocampus and parahippocampal cortex, while the striatum, thalami, brainstem and cerebellum were more spared. A diffuse amyloid angiopathy, in DR112.1 most pronounced in the occipital cortex and cerebellum, was present in all three patients. Hippocampal sclerosis was absent. Isolated α-synuclein immunoreactive Lewy bodies and Lewy neurites were observed in the amygdala of CS770, but absent from CS540. No α-synuclein immunohistochemistry was performed in DR112.1.

### Rare variant association analysis

The frequency of rare PTC mutations and missense variants in *SORL1* was 8.8 % (111 carriers/1255 patients) in the overall patient cohort and 3.7 % (47/1255) for patient-only variants. Mutation frequency in the overall control cohort was 5.6 % (108/1938), and 1.7 % (33/1938) for control-only variants. Mutation frequency in patients with known familial history of AD was 8.9 % (29/327), and 4.0 % (13/327) for patient-only variants.

SKAT-O meta-analysis was performed using all country cohorts except Germany and Czech Republic which did not meet inclusion criteria for association analysis, resulting in a total of *n* = 1085 patients and *n* = 1752 controls. This meta-analysis confirmed significant enrichment of rare PTC mutations and missense variants in patients [SKAT-O *p* value 0.0001; rare allele frequency in patients 5.0 % (108/2170), rare allele frequency in control individuals 2.8 % (98/3504)] (Table 2). Most significant enrichment of these variant in patients was found for the fibronectin type III protein domain (SKAT-O *p* value 0.01) (Supplementary table 8). The fibronectin type III domain is the largest protein domain of the SORL1 protein, spanning amino acids 1527–2108 [37, 44]. The cumulative minor allele frequency was largest for this domain, yet variants were identified in each of the SORL1 functional protein domains (Fig. 1). When excluding PTC variants from this analysis, findings remained the same, with association over the full protein (*p* value 0.0007), and strongest association for fibronectin III domain (*p* value 0.013).

**Table 2:** SKAT-O meta-analysis of rare variant burden

Country of origin	Rare alleles/total alleles patients	Rare alleles/total alleles controls	SKAT-O *p* value
Belgium	27/610 (4.4 %)	38/1488 (2.6 %)	0.01
Spain	34/674 (5.0 %)	11/490 (2.2 %)	0.07
Italy	21/374 (5.6 %)	18/768 (2.3 %)	0.01
Portugal	6/196 (3.1 %)	5/166 (3.0 %)	0.49
Sweden	20/316 (6.3 %)	26/592 (4.4 %)	0.09
Meta-analysis	108/2170 (5.0 %)	98/3504 (2.8 %)	0.0001

Rare variant burden analysis was performed using SKAT-O meta-analysis corrected for gender and *APOE* ε4 status, including individuals originating from Belgium, Spain, Italy, Portugal and Sweden with non-missing information on gender and *APOE* ε4 status. Individuals originating from Germany and Czech Republic were excluded from analysis due to sub-threshold cohort size. Percentages are based on alleles

### Single variant analysis of low-frequency and common variants

We identified six common (MAF ≥ 0.05) variants in the *SORL1* coding sequence, including one missense variant p.Ala528Thr, and five synonymous variants (p.His269His, p.Thr833Thr, p.Ser1187Ser, p.Asn1246Asn, and p.Ala1584Ala). In addition, we identified five low-frequency variants (MAF 0.01–0.05), including three missense variants and two silent variants. Low-frequency missense variant p.Glu270Lys was previously associated with AD in Caribbean-Hispanic familial late-onset AD patients and Northern-European sporadic late-onset AD patients with MAF below 0.01, and was shown to segregate within affected Caribbean-Hispanic families [48]. Fixed-effects meta-analysis showed no significant association for this variant with AD in our cohort [OR 0.75 (95 % CI 0.51–1.12), *p* value 0.17] (Supplementary table 5). Association of missense variant p.Ala528Thr has been demonstrated in Caribbean-Hispanic familial late-onset AD patients at a MAF of 0.16. Fixed-effects meta-analysis showed no significant association for this variant with AD in our cohort [OR 1.22 (95 % CI 0.94–1.59), *p* value 0.14] (Supplementary table 6). Although one synonymous variant showed nominal significance, none of low-frequency and common variants showed significant association with EOAD after correction for multiple testing.

## Discussion

We performed a systematic screening of the complete coding sequence of *SORL1* in a large EOAD patient/control cohort in the frame of the BELNEU and EU EOD consortia. We found an increased burden of rare PTC and non-synonymous variants in the EOAD patients, of whom 8.8 % carried one or more *SORL1* variants. These independent findings corroborated previous reports of an increased frequency of rare *SORL1* variants in EOAD [35, 37]

Strikingly, PTC mutations were exclusively observed in patients. These variants most likely lead to a significant loss of SORL1 protein due to NMD mRNA decay of the mutant transcript. Indeed, we observed reduced SORL1 expression in lymphoblast cells of the p.Gly447Argfs*22 carrier, which increased upon blocking of NMD, indicative of haploinsufficiency. Further, the mode of action of these predicted loss-of-function mutations is in line with the observation of reduced SORL1 expression in post-mortem brain [6] and in human neuronal stable cell lines [1] leading to increased amyloid load. In addition, overexpression of SORL1 cDNA showed decreased amyloid-β secretion in induced human neuronal cells [54]. At a frequency of 0.64 % in the European EOAD cohort, *SORL1* PTC mutations are rare. In familial patients, the frequency of *SORL1* PTC mutations is increased to 1.5 %, which appears in line with reports of *SORL1* PTC mutations in other AD cohorts. Eleven *SORL1* PTC mutations have previously been reported, of which 8 were identified in 484 (1.7 %) familial EOAD patients from France [35, 37] and 3 in 154 (1.9 %) familial LOAD patients of Caribbean-Hispanic origin [48]. We observed a higher frequency of positive family history of AD in PTC variant carriers (71.4 %) compared to carriers of missense variants. Combined with the notion that *SORL1* PTC mutations have not been observed in healthy controls to date, this suggests that PTC variants may have a high disease penetrance, but samples of affected relatives were not available to explore this further. Further evidence is needed to draw inferences on clinical relevance. Compared to carriers of an established pathogenic mutation in one of the three causal genes for EOAD (*PSEN1*, *PSEN2* and *APP*), who had a positive familial history in 92 % of the patients (23/25), familial history of *SORL1* PTC carriers was somewhat lower. In addition, the mean onset age of the *SORL1* PTC carriers (58.6 ± 5.2 years) was higher when compared to the *PSEN1*, *PSEN2* and *APP* carriers (52.3 ± 9.8 years), suggesting a less aggressive disease process. Because our study is limited to EOAD, the upper limit of onset age reported here is determined by the clinical criteria of EOAD, but this does not exclude a role for rare *SORL1* variants in LOAD. In fact, rare *SORL1* variants have previously been associated with familial LOAD by Vardarajan et al. [48].

In contrast to PTC mutations, the frequency of rare missense variants in healthy controls was non-negligible (5.6 %), and included a substantial proportion (69 %) of predicted pathogenic missense variants. This can in part be explained by a lower penetrance of *SORL1* missense variants compared to PTC variants, and/or a variable degree of pathogenic relevance of the identified missense variants for AD. Pathogenicity may differ depending on parameters like the nature of the amino acid substitution, or location of the mutation in specific protein domains, at methylation sites or within adapter protein binding motifs. This necessitates functional follow-up to investigate effects of *SORL1* variants, e.g., on APP trafficking, amyloid-β formation and clearance, to define functional relevance of each rare missense variant. Whereas others have reported that missense variants in *SORL1* may lead to autosomal dominant AD, the relatively high frequency of predicted pathogenic variants in healthy controls in our study indicates that in the absence of functional evidence of pathogenicity, the observation of a *SORL1* missense variant should be interpreted with caution. This caveat notwithstanding, meta-analysis showed a significant enrichment of rare missense variants in patients, which remained significant after exclusion of PTC mutations. This adds to the growing evidence that *SORL1* missense variants may play a role in AD susceptibility. Of note, we obtained evidence of rare variant association in this hypothesis-driven, single gene resequencing study, but in the context of a whole exome sequencing study, this finding would not have survived multiple testing correction, illustrating the need for large sample sizes in hypothesis-free rare variant studies.

We observed missense variants in *SORL1* throughout the different protein coding domains from Vps10p to FANSHY motif, only sparing the propeptide (Fig. 1). One of the missense variants, exclusively found in the patient cohort, p.Gly511Arg, had been detected in two affected relatives of a French autosomal dominant EOAD family [37]. This missense variant was shown to disrupt APP sorting from the trans-Golgi network to the lysosomal degradation pathway through abolished interaction of SORL1 with amyloid-β [7]. We observed p.Gly511Arg in a sporadic patient from Italy with an age at onset of 55 years. We could not perform segregation analysis for this variant due to absence of DNA of relatives. For missense variant p.Tyr1816Cys, located in the fibronectin type III domain, and detected in a patient from Italy with an age at onset of 63 years and a reported familial history of AD, we demonstrated the presence of the variant in an affected relative while absent from an unaffected relative. The elucidation of the crystal structure of the Vps10p and β-propeller domains suggested that amyloid-β monomers are bound by the SORL1 Vps10p domain through beta-sheet interaction, binding amyloid-β inside a tunnel structure formed by a 10-bladed beta-sheet propeller [22]. Rare variants located in the Vps10p domain, such as p.Gly511Arg putatively affect SORL1-amyloid-β interaction by destabilization of the SORL1 beta-sheet structure or disruption of the amyloid-β binding motif. Interestingly, patient-only missense variants affecting the Vps10p domain showed strongest Gibbs free energy changes, indicating strongest effects on SORL1 protein stability.

An alternative functional consequence of rare coding variants involves disruption of the anti-amyloidogenic APP trafficking pathway mediated by SORL1. A binding region for APP at the SORL1 protein is located at the cytoplasmic tail of the protein, where a six amino acid-stretching FANSHY motif is involved in binding the retromer adapter complex. We identified one patient-only missense variant, p.Asn2174Ser, in an Italian sporadic patient with onset age 57 years, altering the third amino acid in the FANSHY sequence from asparagine to serine. The retromer complex functions as the seed of direct interaction of SORL1 with APP. Site-directed mutagenesis disrupting the FANSHY motif in vitro has been shown to be amyloidogenic by ablation of the sequestering of APP by SORL1 to the trans-Golgi network [13, 14].

In contrast with previous investigations of *SORL1* coding variants in late-onset AD cohorts, we could not identify a significant association of common and low-frequency variants (MAF > 0.01) with disease status. Initial association of variants p.Ala528Thr and p.Glu270Lys was reported for familial late-onset cases of Caribbean-Hispanic origin [48]. Discrepancies between variant frequency and direction of effect could be due to cohort ethnicity and founder effects. Absence of significant association for these variants in our EOAD patient/cohort analysis might also reflect reduced pathogenic relevance of these variants in EOAD compared to late-onset AD.

In conclusion, the study we performed represents one of the largest systematic screenings of *SORL1* in EOAD patients and control persons. PTC variants were identified exclusively in patients, and their mode of action corresponds with evidence on the inverse relation between SORL1 expression and amyloid-β formation from in vitro functional studies of SORL1 in AD. The increased proportion of familial disease among PTC variant carriers is indicative of a strong effect on AD pathogenesis. Rare missense variants were associated with increased risk of early-onset AD. Some of these rare missense variants may also exert a strong effect on individual and familial risk of AD. The substantial frequency of (predicted pathogenic) variants in healthy controls, however, necessitates further research on the functional impact of the identified rare *SORL1* variants to elucidate the affected pathways.

## Electronic supplementary material

Below is the link to the electronic supplementary material.

Supplementary material 1 (DOCX 209 kb)

## References

[1] Andersen OM, Reiche J, Schmidt V, Gotthardt M, Spoelgen R, Behlke J, von Arnim CA, Breiderhoff T, Jansen P, Wu X (2005). Neuronal sorting protein-related receptor sorLA/LR11 regulates processing of the amyloid precursor protein. Proc Natl Acad Sci USA.

[2] Andersen OM, Schmidt V, Spoelgen R, Gliemann J, Behlke J, Galatis D, McKinstry WJ, Parker MW, Masters CL, Hyman BT (2006). Molecular dissection of the interaction between amyloid precursor protein and its neuronal trafficking receptor SorLA/LR11. Biochemistry.

[3] Bolger AM, Lohse M, Usadel B (2014). Trimmomatic: a flexible trimmer for Illumina sequence data. Bioinformatics (Oxford, England).

[4] Brouwers N, Sleegers K, Van Broeckhoven C (2008). Molecular genetics of Alzheimer’s disease: an update. Ann Med.

[5] Cacace R, Van den Bossche T, Engelborghs S, Geerts N, Laureys A, Dillen L, Graff C, Thonberg H, Chiang HH, Pastor P (2015). Rare variants in PLD3 do not affect risk for early-onset Alzheimer disease in a European consortium cohort. Human Mutat.

[6] Caglayan S, Bauerfeind A, Schmidt V, Carlo AS, Prabakaran T, Hubner N, Willnow TE (2012). Identification of Alzheimer disease risk genotype that predicts efficiency of SORL1 expression in the brain. Arch Neurol.

[7] Caglayan S, Takagi-Niidome S, Liao F, Carlo AS, Schmidt V, Burgert T, Kitago Y, Fuchtbauer EM, Fuchtbauer A, Holtzman DM (2014). Lysosomal sorting of amyloid-beta by the SORLA receptor is impaired by a familial Alzheimer’s disease mutation. Sci Transl Med.

[8] Campion D, Dumanchin C, Hannequin D, Dubois B, Belliard S, Puel M, Thomas-Anterion C, Michon A, Martin C, Charbonnier F (1999). Early-onset autosomal dominant Alzheimer disease: prevalence, genetic heterogeneity, and mutation spectrum. Am J Hum Genet.

[9] Chen H, Lumley T, Brody J, Heard-Costa NL, Fox CS, Cupples LA, Dupuis J (2014). Sequence kernel association test for survival traits. Genet Epidemiol.

[10] Cuyvers E, De Roeck A, Van den Bossche T, Van Cauwenberghe C, Bettens K, Vermeulen S, Mattheijssens M, Peeters K, Engelborghs S, Vandenbulcke M (2015). Mutations in ABCA7 in a Belgian cohort of Alzheimer’s disease patients: a targeted resequencing study. Lancet Neurol.

[11] Cuyvers E, van der Zee J, Bettens K, Engelborghs S, Vandenbulcke M, Robberecht C, Dillen L, Merlin C, Geerts N, Graff C (2015). Genetic variability in SQSTM1 and risk of early-onset Alzheimer dementia: a European early-onset dementia consortium study. Neurobiol Aging.

[12] Engelborghs S, Dermaut B, Goeman J, Saerens J, Marien P, Pickut BA, Van den Broeck M, Serneels S, Cruts M, Van Broeckhoven C (2003). Prospective Belgian study of neurodegenerative and vascular dementia: APOE genotype effects. J Neurol Neurosurg Psychiatry.

[13] Fjorback AW, Andersen OM (2012). SorLA is a molecular link for retromer-dependent sorting of the Amyloid precursor protein. Commun Integr Biol.

[14] Fjorback AW, Seaman M, Gustafsen C, Mehmedbasic A, Gokool S, Wu C, Militz D, Schmidt V, Madsen P, Nyengaard JR (2012). Retromer binds the FANSHY sorting motif in SorLA to regulate amyloid precursor protein sorting and processing. J Neurosc Off J Soc Neurosci.

[15] Folstein MF, Folstein SE, McHugh PR (1975). “Mini-mental state”. A practical method for grading the cognitive state of patients for the clinician. J Psychiatr Res.

[16] Hermey G (2009). The Vps10p-domain receptor family. Cell Mol Life Sci CMLS.

[17] Hyman BT, Phelps CH, Beach TG, Bigio EH, Cairns NJ, Carrillo MC, Dickson DW, Duyckaerts C, Frosch MP, Masliah E (2012). National Institute on Aging-Alzheimer’s Association guidelines for the neuropathologic assessment of Alzheimer’s disease. Alzheimer’s Dement J Alzheimer’s Assoc.

[18] Prince M, Albanese E, Guerchet M, Prina M (2014) World Alzheimer Report 2014: Dementia and risk reduction: an analysis of protective and modifiable risk factors. Alzheimer's Disease International, London

[19] Jarmolowicz AI, Chen HY, Panegyres PK (2015). The patterns of inheritance in early-onset dementia: Alzheimer’s disease and frontotemporal dementia. Am J Alzheimer’s Dis Other Dement.

[20] Jin C, Liu X, Zhang F, Wu Y, Yuan J, Zhu J, Zhang F, Wang G, Cheng Z (2013). An updated meta-analysis of the association between SORL1 variants and the risk for sporadic Alzheimer’s disease. J Alzheimer’s Dis JAD.

[21] Kim D, Pertea G, Trapnell C, Pimentel H, Kelley R, Salzberg SL (2013). TopHat2: accurate alignment of transcriptomes in the presence of insertions, deletions and gene fusions. Genome Biol.

[22] Kitago Y, Nagae M, Nakata Z, Yagi-Utsumi M, Takagi-Niidome S, Mihara E, Nogi T, Kato K, Takagi J (2015). Structural basis for amyloidogenic peptide recognition by sorLA. Nat Struct Mol Biol.

[23] Koboldt DC, Chen K, Wylie T, Larson DE, McLellan MD, Mardis ER, Weinstock GM, Wilson RK, Ding L (2009). VarScan: variant detection in massively parallel sequencing of individual and pooled samples. Bioinformatics (Oxford, England).

[24] Lambert JC, Ibrahim-Verbaas CA, Harold D, Naj AC, Sims R, Bellenguez C, Jun G, Destefano AL, Bis JC, Beecham GW (2013). Meta-analysis of 74,046 individuals identifies 11 new susceptibility loci for Alzheimer’s disease. Nat Genet.

[25] Lee S, Wu MC, Lin X (2012). Optimal tests for rare variant effects in sequencing association studies. Biostatistics (Oxford, England).

[26] Li H, Durbin R (2009). Fast and accurate short read alignment with Burrows-Wheeler transform. Bioinformatics (Oxford, England).

[27] Livak KJ, Schmittgen TD (2001). Analysis of relative gene expression data using real-time quantitative PCR and the 2(−Delta Delta C(T)) Method. Methods (San Diego, Calif).

[28] McKenna A, Hanna M, Banks E, Sivachenko A, Cibulskis K, Kernytsky A, Garimella K, Altshuler D, Gabriel S, Daly M (2010). The Genome Analysis Toolkit: a MapReduce framework for analyzing next-generation DNA sequencing data. Genome Res.

[29] McKhann G, Drachman D, Folstein M, Katzman R, Price D, Stadlan EM (1984). Clinical diagnosis of Alzheimer’s disease: report of the NINCDS-ADRDA Work Group under the auspices of Department of Health and Human Services Task Force on Alzheimer’s Disease. Neurology.

[30] McKhann GM, Knopman DS, Chertkow H, Hyman BT, Jack CR, Kawas CH, Klunk WE, Koroshetz WJ, Manly JJ, Mayeux R (2011). The diagnosis of dementia due to Alzheimer’s disease: recommendations from the National Institute on Aging-Alzheimer’s Association workgroups on diagnostic guidelines for Alzheimer’s disease. Alzheimer’s Dement J Alzheimer’s Assoc.

[31] McLaren W, Pritchard B, Rios D, Chen Y, Flicek P, Cunningham F (2010). Deriving the consequences of genomic variants with the Ensembl API and SNP Effect Predictor. Bioinformatics (Oxford, England).

[32] Mehmedbasic A, Christensen SK, Nilsson J, Ruetschi U, Gustafsen C, Poulsen AS, Rasmussen RW, Fjorback AN, Larson G, Andersen OM (2015). SorLA complement-type repeat domains protect the amyloid precursor protein against processing. J Biol Chem.

[33] Miyashita A, Koike A, Jun G, Wang LS, Takahashi S, Matsubara E, Kawarabayashi T, Shoji M, Tomita N, Arai H (2013). SORL1 is genetically associated with late-onset Alzheimer’s disease in Japanese. Koreans and Caucasians. PloS One.

[34] Montine TJ, Phelps CH, Beach TG, Bigio EH, Cairns NJ, Dickson DW, Duyckaerts C, Frosch MP, Masliah E, Mirra SS (2012). National Institute on Aging-Alzheimer’s Association guidelines for the neuropathologic assessment of Alzheimer’s disease: a practical approach. Acta Neuropathol.

[35] Nicolas G, Charbonnier C, Wallon D, Quenez O, Bellenguez C, Grenier-Boley B, Rousseau S, Richard AC, Rovelet-Lecrux A, Le Guennec K (2015). SORL1 rare variants: a major risk factor for familial early-onset Alzheimer’s disease. Mol Psychiatry.

[36] Offe K, Dodson SE, Shoemaker JT, Fritz JJ, Gearing M, Levey AI, Lah JJ (2006). The lipoprotein receptor LR11 regulates amyloid beta production and amyloid precursor protein traffic in endosomal compartments. J Neurosci Off J Soc Neurosci.

[37] Pottier C, Hannequin D, Coutant S, Rovelet-Lecrux A, Wallon D, Rousseau S, Legallic S, Paquet C, Bombois S, Pariente J (2012). High frequency of potentially pathogenic SORL1 mutations in autosomal dominant early-onset Alzheimer disease. Mol Psychiatry.

[38] Purcell S, Neale B, Todd-Brown K, Thomas L, Ferreira MA, Bender D, Maller J, Sklar P, de Bakker PI, Daly MJ (2007). PLINK: a tool set for whole-genome association and population-based linkage analyses. Am J Human Genetics.

[39] Reitz C, Cheng R, Rogaeva E, Lee JH, Tokuhiro S, Zou F, Bettens K, Sleegers K, Tan EK, Kimura R (2011). Meta-analysis of the association between variants in SORL1 and Alzheimer disease. Arch Neurol.

[40] Reumers J, De Rijk P, Zhao H, Liekens A, Smeets D, Cleary J, Van Loo P, Van Den Bossche M, Catthoor K, Sabbe B (2012). Optimized filtering reduces the error rate in detecting genomic variants by short-read sequencing. Nat Biotechnol.

[41] Robinson JT, Thorvaldsdottir H, Winckler W, Guttman M, Lander ES, Getz G, Mesirov JP (2011). Integrative genomics viewer. Nat Biotechnol.

[42] Rogaeva E, Meng Y, Lee JH, Gu Y, Kawarai T, Zou F, Katayama T, Baldwin CT, Cheng R, Hasegawa H (2007). The neuronal sortilin-related receptor SORL1 is genetically associated with Alzheimer disease. Nat Genet.

[43] Satya RV, Zavaljevski N, Reifman J (2012). A new strategy to reduce allelic bias in RNA-Seq readmapping. Nucleic acids Res.

[44] Scherzer CR, Offe K, Gearing M, Rees HD, Fang G, Heilman CJ, Schaller C, Bujo H, Levey AI, Lah JJ (2004). Loss of apolipoprotein E receptor LR11 in Alzheimer disease. Arch Neurol.

[45] van der Zee J, Gijselinck I, Dillen L, Van Langenhove T, Theuns J, Engelborghs S, Philtjens S, Vandenbulcke M, Sleegers K, Sieben A (2013). A pan-European study of the C9orf72 repeat associated with FTLD: geographic prevalence, genomic instability, and intermediate repeats. Human Mutat.

[46] van der Zee J, Van Langenhove T, Kovacs GG, Dillen L, Deschamps W, Engelborghs S, Matej R, Vandenbulcke M, Sieben A, Dermaut B (2014). Rare mutations in SQSTM1 modify susceptibility to frontotemporal lobar degeneration. Acta Neuropathol.

[47] Van Durme J, Delgado J, Stricher F, Serrano L, Schymkowitz J, Rousseau F (2011). A graphical interface for the FoldX forcefield. Bioinformatics (Oxford, England).

[48] Vardarajan BN, Zhang Y, Lee JH, Cheng R, Bohm C, Ghani M, Reitz C, Reyes-Dumeyer D, Shen Y, Rogaeva E (2015). Coding mutations in SORL1 and Alzheimer disease. Ann Neurol.

[49] Wang L, Wang S, Li W (2012). RSeQC: quality control of RNA-seq experiments. Bioinformatics (Oxford, England).

[50] Wang Z, Lei H, Zheng M, Li Y, Cui Y, Hao F (2015). Meta-analysis of the Association between Alzheimer Disease and variants in GAB2, PICALM, and SORL1. Mol Neurobiol.

[51] Weckx S, Del-Favero J, Rademakers R, Claes L, Cruts M, De Jonghe P, Van Broeckhoven C, De Rijk P (2005). novoSNP, a novel computational tool for sequence variation discovery. Genome Res.

[52] Willnow TE, Carlo AS, Rohe M, Schmidt V (2010). SORLA/SORL1, a neuronal sorting receptor implicated in Alzheimer’s disease. Rev Neurosci.

[53] Wingo TS, Lah JJ, Levey AI, Cutler DJ (2012). Autosomal recessive causes likely in early-onset Alzheimer disease. Arch Neurol.

[54] Young JE, Boulanger-Weill J, Williams DA, Woodruff G, Buen F, Revilla AC, Herrera C, Israel MA, Yuan SH, Edland SD (2015). Elucidating molecular phenotypes caused by the SORL1 Alzheimer’s disease genetic risk factor using human induced pluripotent stem cells. Cell stem Cell.

