# Supplementary material for: A comprehensive study of the genetic impact of rare variants in SORL1 in European early-onset Alzheimer’s disease
Source: Acta Neuropathol. 2016 Mar 30;132:213–24. doi: 10.1007/s00401-016-1566-9 (PMC4947104; doi:10.1007/s00401-016-1566-9)
Supplement: Supplementary file 1 — Supplementary material 1 (DOCX 209 kb) [file 401_2016_1566_MOESM1_ESM.docx]

**A comprehensive study of the genetic impact of rare variants in *SORL1* in European early-onset Alzheimer's disease**

Jan Verheijen^1,2*^ , Tobi Van den Bossche^1,2,3,4*^, Julie van der Zee^1,2^, Sebastiaan Engelborghs^2,4^, Raquel Sanchez-Valle^5^, Albert Lladó^5^ , Caroline Graff^6,7^, Håkan Thonberg^6,7^, Pau Pastor^8,9^, Sara Ortega-Cubero^9,10^, Maria A. Pastor^10,11,12^, Luisa Benussi^13^, Roberta Ghidoni^13^, Giuliano Binetti^13,14^, Jordi Clarimon^19,15^, Alberto Lleó^9,15^, Juan Fortea^9,15^, Alexandre de Mendonça^16^, Madalena Martins^16^, Oriol Grau-Rivera^17^, Ellen Gelpi^17^, Karolien Bettens^1,2^, Ligia Mateiu^18^, Lubina Dillen^1,2^, Patrick Cras^2,3^, Peter P De Deyn^2,4,†^, Christine Van Broeckhoven^1,2,*^, Kristel Sleegers^1,2,*^, on behalf of the Belgium Neurology (BELNEU) and the European Early-Onset Dementia (EU EOD) Consortia.

**Shared first authors contributed equally*

^1^Neurodegenerative Brain Diseases group, Department of Molecular Genetics, VIB, Antwerp, Belgium
^2^Institute Born-Bunge, University of Antwerp, Antwerp, Belgium

^3^Department of Neurology, Antwerp University Hospital, Edegem, Belgium

^4^Department of Neurology and Memory Clinic, Hospital Network Antwerp (ZNA) Middelheim and Hoge Beuken, Antwerp, Belgium.

^5^Alzheimer's Disease and Other Cognitive Disorders Unit, Neurology Department, Hospital Clínic, Institut d'Investigacions Biomediques August Pi i Sunyer (IDIBAPS), Barcelona, Spain.
^6^Department of Neurobiology, Care Sciences and Society (NVS), Center for Alzheimer Research, Division of Neurogeriatrics, Karolinska Institutet, Huddinge, Sweden.

^7^Department of Geriatric Medicine, Genetics Unit, Karolinska University Hospital, Stockholm, Sweden.

^8^Memory Unit. Department of Neurology, University Hospital Mútua de Terrassa, University of Barcelona School of Medicine, Terrassa, Barcelona, Spain.

^9^Centro de Investigación Biomédica en Red de Enfermedades Neurodegenerativas (CIBERNED), Instituto de Salud Carlos III, Madrid, Spain.

^10^Deparment of Neurology, Complejo Asistencial Universitario de Palencia, Palencia, Spain.

^11^Neuroimaging Laboratory, Division of Neurosciences, Center for Applied Medical Research (CIMA), University of Navarra, Pamplona, Spain.

^12^Department of Neurology, Clínica Universidad de Navarra, University of Navarra School of Medicine, Pamplona, Spain.

^13^Molecular Markers Laboratory, Istituto di Ricovero e Cura a Carattere Scientifico (IRCCS), Istituto Centro San Giovanni di Dio-Fatebenefratelli, Brescia, Italy.

^14^ MAC Memory Center, Istituto di Ricovero e Cura a Carattere Scientifico (IRCCS), Istituto Centro San Giovanni di Dio-Fatebenefratelli, Brescia, Italy

^15^Department of Neurology, IIB Sant Pau, Hospital de la Santa Creu i Sant Pau, Universidad Autònoma de Barcelona, Barcelona, Spain.
^16^Faculty of Medicine and Institute of Molecular Medicine, University of Lisbon, Lisbon, Portugal.

^17^Neurological Tissue Bank of the Biobanc, Hospital Clinic, Institut d'Investigacions Biomediques August Pi i Sunyer (IDIBAPS), Barcelona, Spain

^18^Bioinformatics Unit, Department of Molecular Genetics, VIB, Antwerp, Belgium

*^†^Peter P. De Deyn is also affiliated with the Department of Neurology and Alzheimer Research Center, University of Groningen and University Medical Center Groningen, Groningen, The Netherlands*

**Side author list**

**EU EOD consortium**

Janine Diehl-Schmid, Panagiotis Alexopoulos (Department of Psychiatry and Psychotherapy, Technische Universität München, München, Germany); Benedetta Nacmias, Sandro Sorbi, Silvia Bagnoli (Department of Neurosciences, Psychology, Drug Research and Child Health (NEUROFARBA), University of Florence, Florence, Italy); Maria Rosário Almeida, Isabel Santana (Center for Neuroscience and Cell Biology, University of Coimbra, Coimbra, Portugal); Frederico Simões do Couto (Faculty of Medicine, University of Lisbon, Lisbon, Portugal); Barbara Borroni, Alessandro Padovani (Neurology Unit, University of Brescia, Brescia, Italy); Radoslav Matej, Zdenek Rohan (Center of Clinical Neurosciences, Department of Neurology, First Medical Faculty, Charles University and Department of Pathology and Molecular Medicine, Thomayer Hospital in Prague, Czech Republic); Monica Diez, Cristina Razquin, Elena Lorenzo, Elena Iglesias (Neurogenetics Laboratory, Division of Neurosciences, Center for Applied Medical Research, University of Navarra, Pamplona, Spain); Manuel Seijo-Martínez(Department of Neurology, Hospital do Salnés, Pontevedra, Spain); Ramon Rene, Jordi Gascon, Jaume Campdelacreu (Department of Neurology, Hospital de Bellvitge, Barcelona, Spain); Lena Lilius, Charlotte Forsell, Huei-Hsin Chiang(Department of Neurobiology, Care Sciences and Society (NVS), Center for Alzheimer Research, Division of Neurogeriatrics, Karolinska Institutet, Huddinge, Sweden. Department of Geriatric Medicine, Genetics Unit, Karolinska University Hospital, Stockholm, Sweden)

**BELNEU consortium:**Johan Goeman (Hospital Network Antwerp (ZNA) Middelheim and Hoge Beuken, Antwerp, Belgium), Dirk Nuytten (Hospital Network Antwerp (ZNA) Stuivenberg, Antwerp, Belgium); Mathieu Vandenbulcke, Rik Vandenberghe (University of Leuven and University Hospitals Leuven, Leuven, Belgium); Patrick Santens, Jan De Bleecker, Anne Sieben, Bart Dermaut (University Hospital Ghent, Ghent, Belgium); Jan Versijpt, Alex Michotte (University Hospital Brussels, Brussels, Belgium); Olivier Deryck, Bruno Bergmans (AZ Sint-Jan Brugge, Bruges, Belgium); Christiana Willems (Jessa Hospital, Hasselt, Belgium); Adrian Ivanoiu (Saint-Luc University Hospital, Université Catholique de Louvain, Louvain-la-Neuve, Belgium); and Eric Salmon (University of Liege and Memory Clinic, CHU Liege, Liege, Belgium).

* Corresponding authors:

Prof. Dr. Kristel Sleegers MD PhD

Neurodegenerative Brain Diseases Group

VIB Department of Molecular Genetics, University of Antwerp - CDE

Universiteitsplein 1, B-2610, Antwerp, Belgium

Email: [kristel.sleegers@molgen.vib-ua.be](mailto:kristel.sleegers@molgen.vib-ua.be)

Prof. Dr. Christine Van Broeckhoven PhD DSc

Neurodegenerative Brain Diseases Group

VIB Department of Molecular Genetics, University of Antwerp - CDE

Universiteitsplein 1, B-2610, Antwerp, Belgium

Te. +32 3 265 1101; Fax. +32 3 265 1113

Email: [christine.vanbroeckhoven@molgen.vib-ua.be](mailto:christine.vanbroeckhoven@molgen.vib-ua.be)

**Supplementary information**

**Supplementary figure 1: Pedigree of the p.Tyr1816Cys carrier family.**


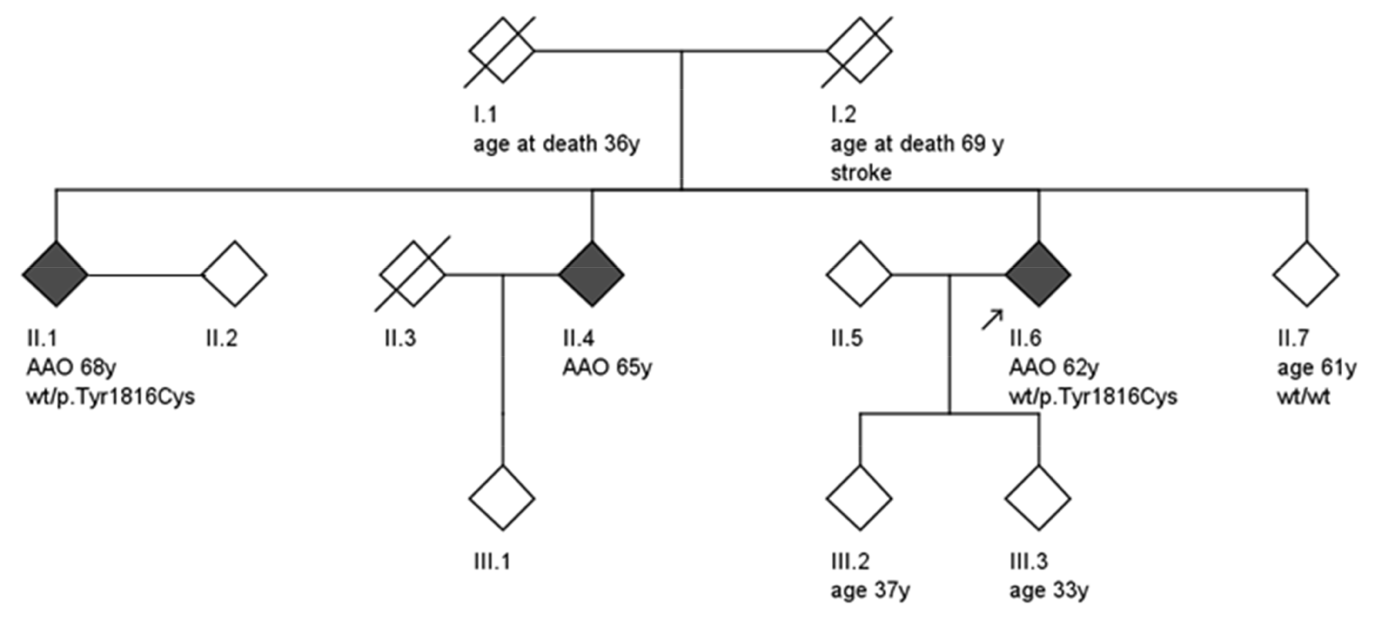


Filled symbols indicate individuals diagnosed with Alzheimer disease. The index case of the pedigree is indicated by a black arrow. DNA was available for family members II.1, II.6 and II.7

| **Supplementary table 1a: Cohort characteristics for each country of origin** | | |
| --- | --- | --- |
| **Country of origin** | **Patients (n=1255)** | **Controls (n=1938)** |
|  |  |  |
| Belgium | n=312 57 % females AAO =63.4 ± 6.1 APOE ε4-postive = 62 % | n=748 60 % females AAI =71.5 ± 9.8 APOE ε4-postive = 26 % |
|  |  |  |
|  |  |  |
| Spain | n=342 59 % females AAO = 57.7 ± 5.1 APOE ε4-postive = 53 % | n=306 58 % females AAI = 58.5 ± 12.8 APOE ε4-postive = 16% |
|  |  |  |
|  |  |  |
| Italy | n=205 67 % females AAO = 57.1 ± 6.9 APOE ε4-postive = 40 % | n=444 58 % females AAI = 64.5 ± 9.5 APOE ε4-postive = 22 % |
|  |  |  |
|  |  |  |
| Portugal | n=106 61 % females AAO = 56.9 ± 6.2 APOE ε4-postive = 37 % | n=130 70.5 % females  AAI = 66.3**±**6.1 APOE ε4-postive = 24 % |
|  |  |  |
|  |  |  |
| Sweden | n=183 62 % females AAO = 58.4 ± 4.8 APOE ε4-postive = 69% | n=303 61 % females AAI = 64 ± 5.4 APOE ε4-postive = 32 % |
|  |  |  |
|  |  |  |
| Germany | n=100 54 % females AAO = 58.5 ± 4.7 APOE ε4-postive = 53 % | n=0 |
|  |  |  |
|  |  |  |
| Czech republic | n=7 29 % females AAO = 56.2 ± 9.4 APOE ε4-postive = 43 % | n=7 29 % females AAI = 59.7 ± 7.8 APOE ε4-postive = 57 % |
|  |  |  |
|  |  |  |

AAO age at onset. AAI age at inclusion

**Supplementary table 1b. Cohort characteristics for each research site**

|  | |  | **N** | **Recruitment** | **Diagnostic / Inclusion criteria** | **Female (%)** | **Age at Onset/Inclusion**  **Mean (years) ± SD** | **Atypical onset (%)** | **Disease Duration**  **Mean (years) ± SD** | **Familial (%)** |
| --- | --- | --- | --- | --- | --- | --- | --- | --- | --- | --- |
| **Belgium** | | | | | | | | | | |
| Flanders-Belgium (K.S.) | AD | | 312 | Hospital-based: Memory Clinic | McKhann et al., 1984 and/or McKhann et al., 2011 | 57.4 | 63.4**±**6.1 | 31.9 | 9.1±4.2 | 43.6 |
|  | control | | 748 | Community-dwelling volunteers and spouses of participants | MMSE >25, MoCA >25, negative history for psychiatric/neurological disorders, negative FH | 60.0 | 71.5**±**9.8 | - | - | - |
| **Spain** | | | | | | | | | | |
| Pamplona (P.P.) | AD | | 180 | Hospital-based: out-clinic | McKhann et al., 1984 | 61.3 | 58.6**±**5.5 | NA | 3.4±1.6 | 49.0 |
|  | control | | 252 | Spouses of out-clinic patients with NBD | Cognitively normal, negative FH | 62.0 | NA | - | - | - |
| Barcelona IDIBAPS (R.S.) | AD | | 73 | University hospital-based | McKhann et al., 2011 | 58.9 | 56.8**±**4.6 | 9.6 | 11.0±0.0 | 47.0 |
|  | control | | 54 | Community-dwelling volunteers and spouses of participants | MMSE >27, normal cognitive test battery, CDR = 0, negative history for psychiatric/neurological disorders | 63.0 | 58.6**±**12.8 | - | - | - |
| Barcelona Sant Pau (J.C.) | AD | | 51 | Hospital-based: Memory Unit | McKhann et al., 1984 | 58.8 | 58.6**±**3.2 | 9.8 | 6.7±3.7 (from first to last clinical visit) | 51.1 |
|  | control | | - | - | - | - | - | - | - | - |
| Barcelona IDIBAPS Brain Bank (E.G.) | AD | | 38 | Brain Bank | Montine et al., 2012 | 45.8 | 54.4**±**4.0 | NA | 11.1±3.4 | 45.8 |
|  | control | | - | - | - | - | - | - | - | - |
| **Italy** | | | | | | | | | | |
| Brescia IRCCS Fatebenefratelli (L.B.) | AD | | 101 | Hospital-based | McKhann et al., 2011 | 64.4 | 59.0**±**6.3 | NA | 5.5±5.7 | 56.8 |
|  | control | | 210 | Spouses and unrelated caregivers of patients | MMSE >26 | 56.7 | 67.8**±**6.3 | - | - | - |
| Florence (B.N.) | AD | | 85 | Hospital-based | DSM-IV | 66.7 | 55.0**±**7.4 | NA | 1.2±0.1 | 15.5 |
|  | control | | 159 | Region-matched volunteers | Neurological disorders excluded | 59.1 | 64.5**±**9.5 | - | - | - |
| Brescia University (B.B.) | AD | | 19 | Hospital-based | McKhann et al., 2011 | 84.2 | 57.0**±**5.2 | NA | 4.6±2.4 | 47.1 |
|  | control | | 75 | Community-dwelling volunteers and spouses | Normal cognitive test battery, negative history for neurological/psychiatric disorders or major illness | 57.3 | 39.2**±**15.2 | - | - | - |
| **Portugal** | | | | | | | | | | |
| Lisbon (A.M.) | AD | | 37 | Hospital and memory-clinic based | McKhann et al., 1984 | 59.5 | 56.0**±**6.0 | NA | 14.0±5.1 | 80.6 |
|  | control | | 130 | Community-dwelling healthy volunteers | CDR = 0, normal (age- and/or education dependent) MMSE and Wechsler logical memory test, normal iADL (Lawton), normal GDS | 70.5 | 66.3**±**6.1 | - | - | - |
| Coimbra (M.R.A.) | AD | | 69 | Hospital-based | McKhann et al., 2011 | 63.2 | 58.0**±**6.3 | NA | NA | NA |
|  | control | | - | - | - | - | - | - | - | - |
| **Czech Republic** | | | | | | | | | | |
| Prague Brain Bank (R.M.) | AD | | 7 | Brain Bank | Montine et al., 2012 | 28.6 | 56.0**±**9.5 | NA | 4.2±1.0 | 25.0 |
|  | control | | 7 | Archived living individuals | Neuropathological confirmed absence of NBD pathology, negative history for neurological/psychiatric disorders, negative FH | 28.6 | 59.7**±**7.8 | - | - | - |
| **Germany** | | | | | | | | | | |
| Munich (J.D.-S.) | AD | | 100 | Hospital-based | McKhann et al., 1984 | 54.0 | 59.0**±**4.7 | NA | NA | NA |
|  | control | | - | - | - | - | - | - | - | - |
| **Sweden** | | | | | | | | | | |
| Stockholm (C.G.) | AD | | 183 | Hospital-based, Geriatric Medicine | McKhann et al., 2011 | 62.1 | 58.0**±**4.8 | 2.2 | 10.6±3.7 | 11.6 |
|  | control | | 303 | Population-based, http://www.snac-k.se/ Lagergren et al., 2004 | >60 years old, MMSE ≥28, neurological disorders excluded | 61.1 | 64.2**±**5.4 | - | - | - |

Note: FH familial history. GDS Geriatric Depression Scale. NBD neurodegenerative brain disease. NA not available. - not applicable.

| **Supplementary table 2: *SORL1* rare coding variants observed in patients** | | | | | | | | | | | |
| --- | --- | --- | --- | --- | --- | --- | --- | --- | --- | --- | --- |
| Protein domain | Genomic level nomenclature | cDNA level nomenclature | Protein level nomenclature | Country of origin | Gender | Family history | Age at onset | Novel variant | SIFT prediction | Mutation Taster prediction | Polyphen prediction |
| Vps10p | g.17821C>T | c.302C>T | p.Ser101Phe | Sweden | f | S | 60 | Novel | Damaging (0) | Disease causing (1) | Probably damaging (1) |
|  | g.25949A>C | c.436A>C | p.Lys146Gln | Italy | f | U | 61 | Novel | Tolerated (0.53) | Disease causing (1) | Benign (0) |
|  | g.44827A>G | c.919A>G | p.Met307Val | Italy | f | U | 59 |  | Damaging (0.02) | Disease causing (1) | Benign (0) |
|  | g.60855C>T | c.994C>T | p.Arg332Trp | Sweden | f | U | 54 |  | Damaging (0) | Disease causing (1) | Probably damaging (1) |
|  | g.60855C>T | c.994C>T | p.Arg332Trp | Spain | f | U | 53 |  | Damaging (0) | Disease causing (1) | Probably damaging (1) |
|  | g.61958_61964delCATCGCAG | c.1050_1057del CATCGCAG | p.Tyr350fs* | Spain | f | S | 65 | Novel | Damaging |  |  |
|  | g.68489C>T | c.1246C>T | p.Arg416* | Sweden | f | U | 55 | Novel |  | Disease causing (1) |  |
|  | g.68579_68579insA | c.1338insA | p.Gly447Argfs*22 | Belgium | m | F | 64 | Novel | Damaging |  |  |
|  | g.70417C>T | c.1438C>T | p.Arg480Cys | Spain | f | F | 63 |  | Damaging (0) | Disease causing (1) | Probably damaging (1) |
|  | g.70501G>A | c.1522G>A | p.Gly508Ser | Italy | f | F | 59 | Novel | Damaging (0) | Disease causing (1) | Probably damaging (1) |
|  | g.70722G>C | c.1531G>C | p.Gly511Arg | Italy | f | S | 55 | Novel | Damaging (0) | Disease causing (1) | Probably damaging (1) |
|  | g.80343G>A | c.1678G>A | p.Glu560Lys | Sweden | f | U | 48 |  | Tolerated (0.09) | Disease causing (1) | Benign (0.27) |
|  | g.93142_93148delCCCCATG | c.1966_1972delCCCCATG | p.Thr659fs*30 | Spain | f | S | 53 | Novel | Damaging |  |  |
|  | g.98455_98457delCT | c.2253_2254delCT | p.Cys752Serfs*22 | Italy | f | F | 50 | Novel | Damaging |  |  |
| β-propeller | g.101753T>C | c.2285T>C | p.Leu762Pro | Belgium | m | S | 68 | Novel | Damaging (0) | Disease causing (1) | Probably damaging (0.99) |
|  | g.101779C>T | c.2311C>T | p.Arg771Cys | Spain | f | S | 54 |  | Damaging (0) | Disease causing (1) | Probably damaging (1) |
|  | g.102999T>G | c.2454T>G | p.Asn818Lys | Spain | f | S | 50 |  | Damaging (0.01) | Disease causing (1) | Probably damaging (1) |
|  | g.105190G>A | c.2650G>A | p.Val884Met | Belgium | m | S | 62 |  | Tolerated (0.27) | Disease causing (1) | Benign (0.01) |
|  | g.106532C>T | c.2807C>T | p.Thr936Met | Italy | f | F | 35 |  | Damaging (0.01) | Disease causing (1) | Probably damaging (1) |
|  | g.106549T>G | c.2824T>G | p.Cys942Gly | Spain | m | S | 63 | Novel | Tolerated (0.41) | Disease causing (1) | Benign (0.41) |
| LDLR class A | g.118037_118037delC | c.3306delC | p.Cys1103Valfs*4 | Spain | f | F | 58 | Novel | Damaging |  |  |
|  | g.125162G>A | c.3544G>A | p.Asp1182Asn | Spain | f | F | 59 | Novel | Damaging (0) | Disease causing (1) | Probably damaging (1) |
|  | g.134059G>C | c.3746G>C | p.Cys1249Ser | Spain | m | U | 62 |  | Damaging (0) | Disease causing (1) | Probably damaging (1) |
|  | g.135830C>T | c.3827C>T | p.Thr1276Met | Germany | f | U | 52 |  | Damaging (0.01) | Disease causing (1) | Probably damaging (1) |
|  | g.137140T>C | c.4030T>C | p.Cys1344Arg | Sweden | f | U | 49 | Novel | Damaging (0) | Disease causing (1) | Probably damaging (1) |
|  | g.138810C>T | c.4225C>T | p.Leu1409Phe | Belgium | f | U | 61 |  | Tolerated (0.71) | Benign (0.93) | Benign (0) |
|  | g.138909C>T | c.4324C>T | p.Arg1442* | Portugal | f | F | 61 | Novel |  | Disease causing (1) |  |
|  | g.138924G>A | c.4339G>A | p.Gly1447Ser | Spain | m | U | 60 | Novel | Damaging (0.08) | Disease causing (1) | Possibly damaging (0.91) |
|  | g.138943G>C | c.4358G>C | p.Cys1453Ser | Spain | f | S | 61 | Novel | Damaging (0.01) | Disease causing (1) | Probably damaging (1) |
|  | g.138945C>T | c.4360C>T | p.Pro1454Ser | Sweden | f | U | 45 |  | Tolerated (0.45) | Disease causing (1) | Probably damaging (1) |
|  | g.138945C>T | c.4360C>T | p.Pro1454Ser | Sweden | f | U | 58 |  | Tolerated (0.45) | Disease causing (1) | Probably damaging (1) |
|  | g.143499G>T | c.4449G>T | p.Thr1483Met | Belgium | f | U | 69 |  | Damaging (0.16) | Benign (1) | Possibly damaging (0.68) |
|  | g.152035G>A | c.4564G>A | p.Glu1522Lys | Italy | f | S | 57 | Novel | Tolerated (0.37) | Disease causing (0.99) | Possibly damaging (0.47) |
| fibronectin type III | g.152113G>A | c.4642G>A | p.Ala1548Thr | Spain | m | U | 51 | Novel | Tolerated (0.23) | Benign (1) | Benign (0.02) |
|  | g.153201G>A | c.4780G>A | p.Val1594Met | Sweden | m | U | 56 |  | Tolerated (0.19) | Benign (0.98) | Benign (0.01) |
|  | g.155930G>C | c.5195G>C | p.Gly1732Ala | Sweden | f | U | 65 |  | Tolerated (0.32) | Disease causing (1) | Probably damaging (0.99) |
|  | g.155974_155974delG | c.5241delG | p.Val1747fs* | Belgium | f | F | 64 |  | Neutral |  |  |
|  | g.162696A>G | c.5447A>G | p.Tyr1816Cys | Italy | m | F | 63 |  | Damaging (0) | Disease causing (1) | Probably damaging (1) |
|  | g.162696A>G | c.5447A>G | p.tyr1816Cys | Spain | m | F | 59 |  | Damaging (0) | Disease causing (1) | Probably damaging (1) |
|  | g.167690C>T | c.5864C>T | p.Pro1955Leu | Italy | f | F | 64 |  | Damaging (0) | Disease causing (1) | Probably damaging (1) |
|  | g.167699C>G | c.5873C>G | p.Ser1958Cys | Germany | f | U |  |  | Damaging (0.01) | Disease causing (1) | Possibly damaging (0.93) |
|  | g.169012G>A | c.6040G>A | p.Asp2014Asn | Italy | f | S | 54 | Novel | Tolerated (0.19) | Disease causing (1) | Possibly damaging (0.72) |
|  | g.170007C>G | c.6112C>G | p.His2038Asp | Spain | f | F | 62 |  | Tolerated (0.94) | Disease causing (1) | Probably damaging (0.99) |
|  | g.172979G>T | c.6268G>T | p.Gly2090Cys | Spain | f | U | 60 | Novel | Damaging (0.01) | Disease causing (1) | Probably damaging (1) |
|  | g.172979G>T | c.6269G>T | p.Gly2090Val | Spain | m | S | 63 |  | Damaging (0.01) | Disease causing (1) | Probably damaging (1) |
| cytosolic | g.175429A>G | c.6441A>G | p.Ile2147Met | Spain | f | U | 65 |  | Damaging (0.02) | Benign (0.86) | Benign (0.26) |
|  | g.175509A>G | c.6521A>G | p.Asn2174Ser | Italy | f | S | 57 | Novel | Damaging (0.11) | Disease causing (1) | Probably damaging (1) |

Note: Genomic DNA level nomenclature was based on NC_000011.9, cDNA level nomenclature was based on NM_003105 according to hg19/GRCh37. Protein level nomenclature was based on NP_003096. Novelty status of variants was based on absence of the variant in the Database of Single Nucleotide Polymorphisms 141, the Exome Variant Server, the International HapMap Project, the 1000 Genomes Project and the Exome Aggregation Consortium database. AD Alzheimer’s disease. Prob probable. S sporadic U unknown F familial.

**Supplementary table 3: *SORL1* rare coding variants observed in patients and controls**

| Protein domain | Genomic level nomenclature | cDNA level nomenclature | Protein level nomenclature | Control carriers | Patient carriers |
| --- | --- | --- | --- | --- | --- |
|  |  |  |  |  |  |
| Vps10p | g.25931C>A | c.420C>A | p.Asp140Asn | 1 | 1 |
|  | g.62020A>C | c.1112A>C | p.Asn371Thr | 6 | 4 |
|  | g.93082T>A | c.1906T>A | p.Ser636Thr | 2 | 3 |
| β-propeller | g.98402G>A | c.2200G>A | p.Asp734Asn | 3 | 4 |
|  | g.98450G>A | c.2248G>A | p.Val750Ile | 6 | 2 |
| LDLR class A | g.114908C>G | c.3220C>G | p.Gln1074Glu | 1 | 1 |
|  | g.118068C>T | c.3337C>T | p.Pro1113Ser | 1 | 1 |
|  | g.135935C>G | c.3932C>G | p.Ala1311Gly | 1 | 1 |
|  | g.138888A>T | c.4303A>T | p.Thr1435Ser | 3 | 4 |
|  | g.143519C>T | c.4468C>T | p.Arg1490Cys | 1 | 1 |
| fibronectin type III | g.152948G>C | c.4689G>C | p.Trp1563Cys | 3 | 1 |
|  | g.154663C>T | c.4969C>T | p.Leu1657Phe | 1 | 1 |
|  | g.155013G>A | c.5091G>A | p.Met1697Ile | 2 | 1 |
|  | g.162675A>G | c.5426A>G | p.Asn1809Ser | 5 | 3 |
|  | g.162687T>A | c.5439T>A | p.His1813Gln | 24 | 22 |
|  | g.162687A>G | c.5438A>G | p.His1813Arg | 1 | 1 |
|  | g.172905A>C | c.6194A>C | p.Asp2065Val | 12 | 11 |
|  | g.172959A>G | c.6248A>G | p.Lys2083Arg | 1 | 1 |
|  | g.173000G>A | c.6289G>A | p.Val2097Ile | 3 | 5 |

Genomic DNA level nomenclature was based on NC_000011.9, cDNA level nomenclature was based on NM_003105 according to hg19/GRCh37. Protein level nomenclature was based on NP_003096.

**Supplementary table 4: *SORL1* rare coding variants observed in controls**

| Protein domain | genomic level nomenclature | cDNA level nomenclature | Protein level nomenclature | Country of origin | Gender | Age at inclusion | Novel variant | SIFT prediction | Mutation Taster prediction | Polyphen prediction |
| --- | --- | --- | --- | --- | --- | --- | --- | --- | --- | --- |
|  |  |  |  |  |  |  |  |  |  |  |
| Vps10p | g.35915G>A | c.614G>A | p.Arg205Gln | Sweden | m | 60 |  | Tolerated (0.19) | Disease causing (1) | Possibly damaging (0.91) |
|  | g.60831G>C | c.970G>C | p.Val324Leu | Belgium | m | 79 |  | Tolerated (0.94) | Disease causing (1) | Probably damaging (1) |
|  | g.91389T>C | c.1729T>C | p.Ser577Pro | Belgium | m | 62 |  | Damaging (0.09) | Disease causing (1) | Probably damaging (1) |
|  | g.93092G>A | c.1916G>A | p.Arg639Gln | Portugal | m |  |  | Tolerated (0.49) | Disease causing (1) | Probably damaging (1) |
|  | g.93100G>A | c.1924G>A | p.Glu642Lys | Italy | f | 55 | Novel | Tolerated (0.28) | Disease causing (1) | Probably damaging (0.97) |
|  | g.93197A>G | c.2021A>G | p.Asn674Ser | Belgium | f | 63 |  | Tolerated (0.7) | Disease causing (1) | Probably damaging (1) |
|  | g.98430C>T | c.2228C>T | p.Ala743Val | Belgium | f | 74 |  | Tolerated (0.29) | Disease causing (1) | Benign (0) |
|  | g.98430C>T | c.2228C>T | p.Ala743Val | Belgium | f | 86 |  | Tolerated (0.29) | Disease causing (1) | Benign (0) |
| β-propeller | g.101888T>C | c.2420T>C | p.Leu807Pro | Belgium | m | 75 | Novel | Damaging (0.01) | Disease causing (1) | Probably damaging (0.98) |
|  | g.103099G>C | c.2554G>C | p.Gly852Arg | Portugal |  |  | Novel | Damaging (0.35) | Disease causing (1) | Probably damaging (1) |
|  | g.105148G>A | c.2608G>A | p.Val870Ile | Spain | f | 53 |  | Tolerated (0.09) | Disease causing (1) | Possibly damaging (0.82) |
|  | g.105193C>A | c.2653C>A | p.Pro885Thr | Italy | f |  |  | Damaging (0.04) | Disease causing (1) | Benign (0.3) |
|  | g.107345A>G | c.2939A>G | p.Gln980Arg | Belgium | f | 83 | Novel | Tolerated (0.59) | Disease causing (1) | Benign (0) |
|  | g.107411C>T | c.3005C>T | p.Thr1002Met | Belgium | f | 65 |  | Damaging (0.06) | Disease causing (1) | Probably damaging (1) |
|  | g.107411C>T | c.3005C>T | p.Thr1002Met | Belgium | f | 81 |  | Damaging (0.06) | Disease causing (1) | Probably damaging (1) |
| LDLR class A | g.117982G>A | c.3251G>A | p.Arg1084His | Belgium | m | 75 |  | Tolerated (0.08) | Disease causing (1) | Benign (0.03) |
|  | g.134114T>A | c.3801T>A | p.Asp1267glu | Spain | f |  | Novel | Damaging (0) | Disease causing (1) | Probably damaging (1) |
|  | g.135874T>C | c.3871T>C | p.Phe1291Leu | Italy | f | 57 |  | Tolerated (0.69) | Disease causing (1) | Benign (0.03) |
|  | g.135895G>A | c.3892G>A | p.Gly1298Arg | Spain | f | 50 |  | Damaging (0.1) | Disease causing (1) | Probably damaging (1) |
|  | g.135932C>T | c.3929C>T | p.Ala1310Val | Sweden | f | 61 |  | Tolerated (0.26) | Disease causing (1) | Possibly damaging (0.67) |
|  | g.135932C>T | c.3929C>T | p.Ala1310Val | Sweden | m | 67 |  | Tolerated (0.26) | Disease causing (1) | Possibly damaging (0.67) |
|  | g.137074C>A | c.3964C>A | p.His1322Asn | Belgium | m | 79 |  | Tolerated (0.66) | Disease causing (1) | Benign (0.1) |
|  | g.137895G>A | c.4136G>A | p.Gly1379Asp | Sweden | f | 66 |  | Damaging (0.18) | Disease causing (1) | Probably damaging (1) |
|  | g.138903G>C | c.4318G>C | p.Gly1440Ser | Italy | f |  |  | Damaging (0) | Disease causing (1) | Probably damaging (1) |
|  | g.143460G>T | c.4409G>T | p.Arg1470Leu | Sweden | f | 61 |  | Damaging (0.37) | Disease causing (0.83) | Benign (0) |
|  | g.143493C>T | c.4442C>T | p.Pro1481Leu | Sweden | f | 61 |  | Tolerated (0.41) | Disease causing (1) | Benign (0.1) |
|  | g.143562C>A | c.4511C>A | p.Ala1504Asp | Sweden | f | 60 | Novel | Tolerated (0.63) | Benign (0.57) | Benign (0.07) |
|  | g.152100G>C | c.4629G>C | p.Glu1543Asp | Belgium | m | 82 |  | Tolerated (0.28) | Benign (0.91) | Benign (0.12) |
|  | g.152100G>C | c.4629G>C | p.Glu1543Asp | Belgium | f | 70 |  | Tolerated (0.28) | Benign (0.91) | Benign (0.12) |
| fibronectin type III | g.154696G>A | c.5002G>A | p.Val1668Met | Italy | f | 54 |  | Damaging (0.22) | Benign (0.73) | Probably damaging (0.99) |
|  | g.166620C>T | c.5653C>T | p.Pro1885Ser | Italy | f |  | Novel | Tolerated (0.75) | Disease causing (1) | Probably damaging (0.99) |
|  | g.167555G>A | c.5729G>A | p.Arg1910His | Belgium | m | 78 |  | Tolerated (0.13) | Disease causing (1) | Probably damaging (1) |
|  | g.168871G>A | c.5899G>A | p.Val1967Ile | France | f |  |  | Tolerated (1) | Benign (0) | Benign (0) |

Genomic DNA level nomenclature was based on NC_000011.9, cDNA level nomenclature was based on NM_003105 according to hg19/GRCh37. Protein level nomenclature was based on NP_003096. Novelty status of variants was based on absence of the variant in the Database of Single Nucleotide Polymorphisms 141, the Exome Variant Server, the International HapMap Project, the 1000 Genomes Project and the Exome Aggregation Consortium database.

**Supplementary table 5: Low-frequency (MAF 0.01 – 0.05) CDS variants identified in patients and controls**

| Variant | dbSNP | Protein domain | MAF patients | MAF controls | MAF EVS | Fixed-effect meta-analysis | |
| --- | --- | --- | --- | --- | --- | --- | --- |
|  |  |  |  |  |  | OR (95% CI) | p-value |
| p.Glu270Lys | rs117260922 | Vps10p | 0.02 | 0.03 | 0.01 | 0.75 (0.51 – 1.12) | 0.17 |
| p.Phe1099Leu | rs146903951 | LDLR class A | 0.02 | 0.03 | 0.01 | 0.73 (0.41 – 1.32) | 0.3 |
| p.Ile1116Val | rs62617129 | LDLR class A | 0.01 | 0.01 | 0.01 | 0.93 (0.46 – 1.9) | 0.85 |
| p.Asn1392Asn | rs2276412 | LDLR class A | 0.02 | 0.02 | 0.02 | 0.76 (0.48 – 1.18) | 0.22 |
| p.Lys1895Lys | rs17125548 | fibronectin type III | 0.03 | 0.03 | 0.03 | 1.04 (0.92 – 1.18) | 0.52 |

EVS Exome variant server. OR odds ratio. CI confidence interval. MAF minor allele frequency. MAF EVS minor allele frequency as reported by the Exome variants server webpage (http://evs.gs.washington.edu/EVS/).

**Supplementary table 6: Common (MAF ≥ 0.05) CDS variants identified in patients and controls**

| Variant | dbSNP | Protein domain | MAF patients | MAF controls | MAF EVS | Fixed-effect meta-analysis | |
| --- | --- | --- | --- | --- | --- | --- | --- |
|  |  |  |  |  |  | OR (95% CI) | p-value |
| p.His269His | rs12364988 | Vps10p | 0.51 | 0.47 | 0.48 | 1.12 (1.0 - 1.25) | 0.06 |
| p.Ala528Thr | rs2298813 | Vps10p | 0.06 | 0.05 | 0.05 | 1.22 (0.94 – 1.59) | 0.14 |
| p.Thr833Thr | rs78274293 | β-propeller | 0.07 | 0.08 | 0.07 | 0.78 (0.63 – 0.98) | 0.03 |
| p.Ser1187Ser | rs2070045 | LDLR class A | 0.23 | 0.22 | 0.23 | 1.1 (0.92 – 1.21) | 0.44 |
| p.Asn1246Asn | rs1699102 | LDLR class A | 0.33 | 0.33 | 0.33 | 1.04 (0.92 – 1.19) | 0.51 |
| p.Ala1584Ala | rs3824968 | fibronectin type III | 0.31 | 0.3 | 0.31 | 1.04 (0.92 – 1.18) | 0.5 |

EVS Exome variant server. OR odds ratio. CI confidence interval. MAF minor allele frequency. MAF EVS minor allele frequency as reported by the Exome variants server webpage (http://evs.gs.washington.edu/EVS/).

**Supplementary table 7: Effects of missense variants in the Vps10p domain on Gibbs free energy changes**

| Cohort | Variant | Gibbs free energy change (∆∆G) |
| --- | --- | --- |
| patient | p.Ser101Phe | 0.03 |
|  | p.Lys146Gln | 0.49 |
|  | p.Mer307Val | 1.21 |
|  | p.Ser332Trp | 5 |
|  | p.Ser480Cys | N.A. |
|  | p.Gly508Ser | 10.59 |
|  | p.Gly511Arg | 2.5 |
|  | p.Glu560Lys | 0.19 |
| control | p.Arg205Gln | 0.99 |
|  | p.Val324Leu | -0.52 |
|  | p.Ser577Pro | 6.6 |
|  | p.Arg639Gln | -0.02 |
|  | p.Glu642Lys | -0.56 |
|  | p.Asn674Ser | 0.48 |
|  | p.Ala743Val | 0.38 |
| patient and control | p.Asp140Asn | 0.65 |
|  | p.Asn371Thr | 0.19 |
|  | p.Ser636Thr | 1.5 |

A positive Gibbs free energy change value (∆∆G) indicates reduced protein stability. N.A. not available.

**Supplementary table 8: SKAT-O meta-analysis of rare variant burden separate for each SORL1 functional protein domain.**

| Protein domain | Belgium | | |  | Spain | | |  | Italy | | |  | Portugal | | |  | Sweden | | |  | All countries of origin | | |
| --- | --- | --- | --- | --- | --- | --- | --- | --- | --- | --- | --- | --- | --- | --- | --- | --- | --- | --- | --- | --- | --- | --- | --- |
|  | RAF patients | RAF controls | p-value |  | RAF patients | RAF controls | p-value |  | RAF patients | RAF controls | p-value |  | RAF patients | RAF controls | p-value |  | RAF patients | RAF controls | p-value |  | RAF patients | RAF controls | p-value |
|  |  |  |  |  |  |  |  |  |  |  |  |  |  |  |  |  |  |  |  |  |  |  |  |
| Vps10p | 3/610 (0.5%) | 7/1488 (0.5%) | 0.34 |  | 4/674 (0.6%) | 0/490 | 0.09 |  | 5/374 (1.3%) | 2/768 (0.3%) | 0.08 |  | 1/196 (0.5%) | 2/166 (1.2%) | 0.39 |  | 6/316 (1.9%) | 6/592 (1.0%) | 0.2 |  | 19/2186 (0.9%) | 17/3504 (0.5%) | 0.02 |
|  |  |  |  |  |  |  |  |  |  |  |  |  |  |  |  |  |  |  |  |  |  |  |  |
| β-propeller | 3/610 (0.5%) | 5/1488 (0.3%) | 0.37 |  | 4/674 (0.6%) | 2/490 (0.4%) | 0.35 |  | 4/374 (1.1%) | 2/768 (0.3%) | 0.1 |  | 0/196 | 0/166 | 1 |  | 4/316 (1.3%) | 3/592 (0.5%) | 0.69 |  | 15/2186 (0.7%) | 12/3504 (0.3%) | 0.19 |
|  |  |  |  |  |  |  |  |  |  |  |  |  |  |  |  |  |  |  |  |  |  |  |  |
| LDLR class A | 3/610 (0.5%) | 7/1488 (0.5%) | 0.54 |  | 8/674 (1.2%) | 4/490 (0.4%) | 0.4 |  | 3/374 (0.8%) | 3/768 (0.4%) | 0.22 |  | 2/196 (1.0%) | 1/166 (0.5%) | 0.17 |  | 2/316 (0.6%) | 6/592 (1.0%) | 0.16 |  | 18/2186 (0.8%) | 21/3504 (0.6%) | 0.22 |
|  |  |  |  |  |  |  |  |  |  |  |  |  |  |  |  |  |  |  |  |  |  |  |  |
| Fibronectin type III | 18/610 (3.0%) | 19/1488 (1.3%) | 0.02 |  | 17/674 (2.5%) | 5/490 (1%) | 0.28 |  | 8/374 (2.2%) | 11/768 (1.6%) | 0.44 |  | 3/196 (1.5%) | 2/166 (1.2%) | 0.32 |  | 8/316 (2.5%) | 11/592 (1.9%) | 0.28 |  | 54/2186 (2.4%) | 48/3504 (1.4%) | 0.005 |
|  |  |  |  |  |  |  |  |  |  |  |  |  |  |  |  |  |  |  |  |  |  |  |  |
| cytosolic tail | 0/610 | 0/1488 | 1 |  | 1/674 (0.1%) | 0/490 | 0.68 |  | 1/374 (0.3%) | 0/768 | 0.13 |  | 0/196 | 0/166 | 1 |  | 0/316 | 0/592 | 1 |  | 2/2186 (0.09%) | 0/3504 | 0.17 |
|  |  |  |  |  |  |  |  |  |  |  |  |  |  |  |  |  |  |  |  |  |  |  |  |
| Full protein | 27/610 (4.4%) | 38/1488 (2.6%) | 0.01 |  | 34/674 (5.0%) | 11/490 (2.2%) | 0.07 |  | 21/374 (5.6%) | 18/768 (2.3%) | 0.01 |  | 6/196 (3.1%) | 5/166 (3.0%) | 0.49 |  | 20/316 (6.3%) | 26/592 (4.4%) | 0.09 |  | 108/2170 (5.0%) | 98/3504 (2.8%) | 0.0001 |
|  |  |  |  |  |  |  |  |  |  |  |  |  |  |  |  |  |  |  |  |  |  |  |  |

Burden analysis was performed using SKAT-O meta-analysis corrected for gender and APOE ԑ4 status, including individuals originating from Belgium, Spain, Italy, Portugal and Sweden. Correction for multiple testing was performed using Šidák correction. RAF rare allele frequency.
